# A study of gender-specific differences in online platform in medical education

**DOI:** 10.1186/s12909-026-10010-6

**Published:** 2026-07-25

**Authors:** Maëlle Moranges, Noha Ibrahim, Sihem Amer-Yahia, Olivier Palombi

**Affiliations:** Computer Science Laboratory of Grenoble, CNRS, Université Grenoble Alpes, Saint-Martin-d’Hères, 38400 France

**Keywords:** Undergraduate medicine, Online learning, Student engagement, Assessment and performance, Educational equity, Gender differences, Subgroup Discovery, Pattern mining, Artificial intelligence

## Abstract

**Background:**

This study explores gender-specific learning patterns among medical students using a national online educational platform in France. Although the proportion of women admitted to medical schools has steadily increased, persistent gender disparities remain in medical career progression. These inequalities may already emerge during university training, influencing learning behaviors. Understanding when and how these differences appear is crucial to designing targeted interventions that promote equity in medical education.

**Methods:**

We analyzed anonymized data from all sixth-year medical students in France during the 2022–2023 and 2023–2024 academic years. This dataset covers the entire national cohort using the online platform to prepare for mandatory medical examinations. We applied a *Subgroup Discovery* algorithm using gender as the target variable to identify interpretable patterns associated with online engagement, learning activity and academic performance that characterize male and female students. Analyses considered platform use, participation in interactive activities, and performance across assessment formats and medical specialties.

**Results:**

Our analysis revealed marked gender differences in both engagement and academic performance. Male students were more likely to belong to subgroups characterized by high engagement with the platform or higher academic performance, particularly in Progressive Case assessments and several medical specialties. Female students were more frequently associated with lower participation in the platform’s interactive features and with response patterns suggesting a greater tendency to omit correct answer choices. Together with the existing literature, these findings generated several hypotheses regarding the mechanisms underlying these disparities, including assessment design, response strategies under uncertainty, examination context, and psychological factors.

**Conclusions:**

Gender disparities in performance are already observable during medical training, before students enter professional practice. These findings highlight the importance of evaluating how assessment design, online learning environments, and educational support influence gender equity in medical education. This study also illustrates the value of data-driven approaches such as *Subgroup Discovery* for generating testable hypotheses that can inform future educational research and interventions.

**Supplementary Information:**

The online version contains supplementary material available at https://doi.org/10.1186/s12909-026-10010-6.

## Background

Over the past few decades, the proportion of women enrolled in medical programs has steadily increased. Nonetheless, gender inequalities persist in medical careers, with women remaining underrepresented in leadership positions [1, 2] and in certain specialties [3]. These disparities are partly attributed to family responsibilities [4] and insufficient professional support [5]. Importantly, these inequalities may emerge early during medical education, raising questions about how medical students learn and prepare for their future profession.

A growing body of literature has examined how gender disparities manifest during medical training. Several studies have documented gender-based discrimination in performance evaluations of residents, particularly in internal medicine [6], emergency medicine [7, 8], surgery [6], and gynecology [9]. These disparities are often linked to implicit biases [10–12], conflicting professional expectations [8, 13], and inconsistent feedback provided to women [13]. Some studies report that female residents initially outperform men on key competencies but then progress at a slower rate [14]. Others highlight differences in empathy [15] and emotional intelligence [16], which may influence learning and performance. However, findings remain mixed, as several studies have reported no significant gender bias [17–19].

Most of these studies rely on qualitative analyses [8, 12, 20, 21] or traditional statistical methods such as regression models [9, 17, 18], hierarchical models [7, 14, 19], or ANOVA [15]. While these approaches provide valuable insights, they often fail to fully capture the complexity and heterogeneity of learners’ behaviors, performance trajectories, study strategies, and interactions.

Artificial intelligence methods are increasingly used in education research, particularly in online contexts. They have been applied to predict student performance in mathematics [22], computer science [23, 24], biology [25], and medicine [26, 27], often examining the influence of platform design [23], resource usage [22, 26], and engagement with learning activities [24, 25]. However, these studies typically focus on a single course over a limited period, overlook inter-individual dynamics, and rarely account for demographic characteristics such as gender. Many employ supervised machine learning algorithms, including artificial neural networks [26, 27], random forests [25], or ensemble models [24]. Although some studies use explainability methods [24], most models remain black boxes with limited interpretability. Descriptive AI approaches are rarely employed in this context; only clustering [22] and sequence pattern analysis [23] have been explored to date. While artificial intelligence methods have expanded the analytical toolkit, they often prioritize predictive accuracy over interpretability, making it difficult to translate findings into concrete educational strategies. To our knowledge, no study has yet combined online behavioral data with an interpretable analytical frameworks to systematically investigate differences between populations in medical education.

In this context, the French medical education system offers an ideal setting to address these gaps. Since 2016, national medical examinations have been fully digital, and students across all 32 medical faculties use a shared online platform (*uness.fr*) to prepare for these assessments. This platform integrates the National Training Bank (NTB), which provides standardized learning materials and mock exams for the national ranking tests (ECNi until 2023, then EDN). The ECNi (computerized National Ranking Examination) consisted solely of multiple-choice questions (MCQs), including single-best-answer questions and multiple-answer questions, organized into three examination components: Progressive Cases (PC), Critical Article Reading (CAR), and Isolated Question Sequences (IQS). The EDN (National Digital Examinations) retain these components while introducing additional response formats, including Short Open-Ended Response Questions, Image-Based Pointing Questions, and Specified Response Questions, in which candidates know in advance the exact number of responses to select. Future examinations will also include Script Concordance Tests, which assess how newly available clinical information modifies the probability of diagnostic or management hypotheses using a Likert scale ranging from *-2* to 2. The EDN also introduce a new clinical scenario, the Key Feature Problem (KFP). KFPs focus on key clinical decisions and assess clinical reasoning using the previously described response formats. Finally, the EDN are complemented by a separate practical assessment, the Objective Structured Clinical Examination (OSCE), which evaluates clinical and communication skills in standardized simulated clinical encounters. Exam performance determines access to internships and specialties, thereby shaping students’ career paths. Consequently, the platform records detailed longitudinal data on students’ engagement and performance, creating unprecedented opportunities to analyze learning behaviors at a national scale.

Building on these opportunities, our study investigates potential gender-based differences in online learning behaviors among French medical students. We analyze data from two national cohorts of sixth-year students (2022–2023 and 2023–2024) using a data exploration methodology based on the *Subgroup Discovery* algorithm [28]. This approach is particularly suited for uncovering interpretable patterns and subgroup-specific behaviors within large, heterogeneous datasets. Applied to online learning traces, it enables the identification of behavioral differences between male and female students and provides actionable insights for educational strategies.

Gender is treated in a binary manner (male/female) due to data constraints (civility titles and first names; see Section A in Additional file), but the methodology can easily accommodate more inclusive gender categories in future work. The objective is to identify significant gender disparities and inform targeted interventions that promote more equitable and inclusive medical education.

**Fig. 1:**
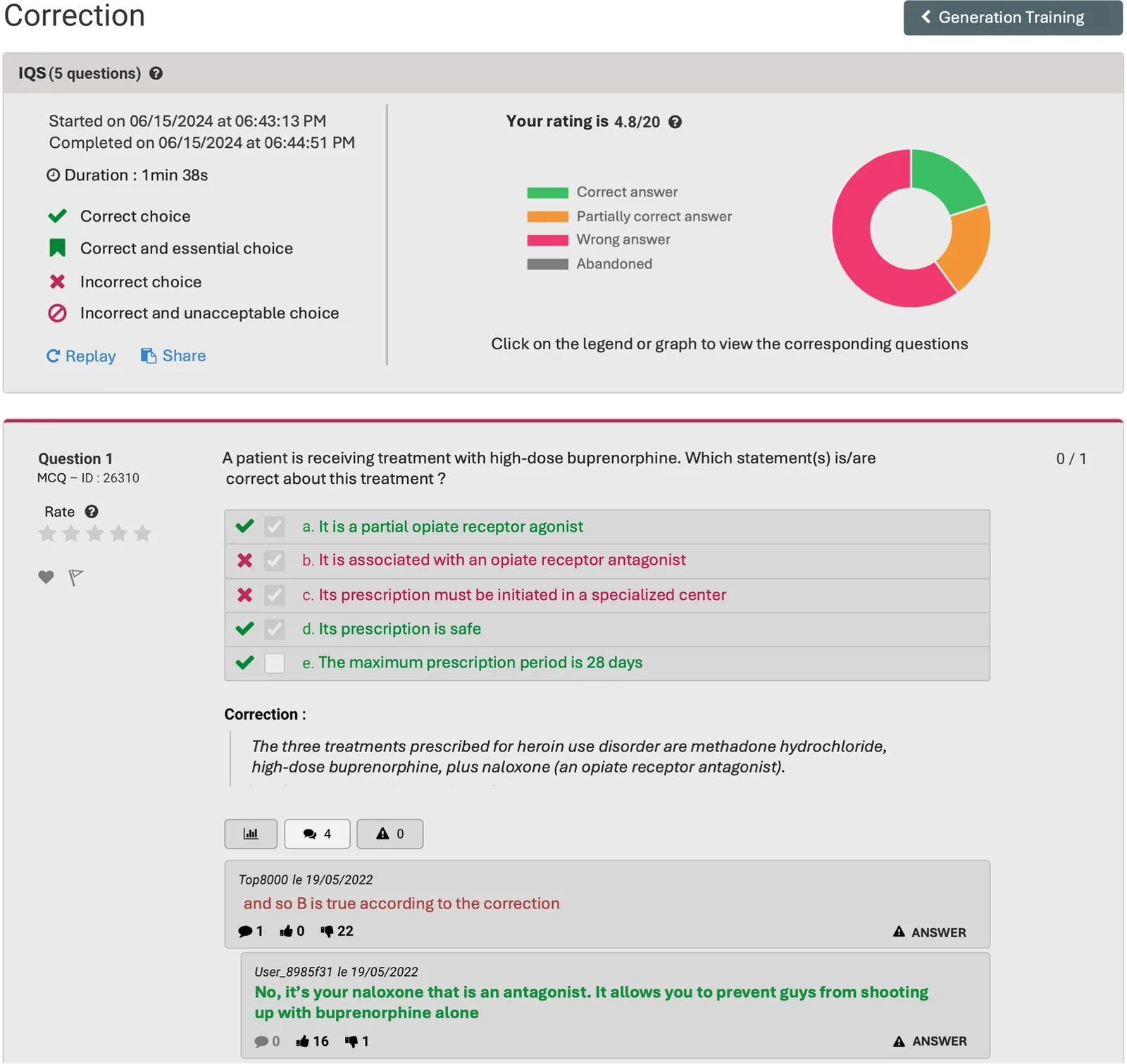
Overview of the initial section of an IQS correction page in the NTB platform (translated into English by the authors). The upper panel summarizes the student’s performance for the completed session, including the overall score, distribution of correct and incorrect answers, and temporal information. The lower panel displays the first question with the student’s selected answers, the official correction and explanatory feedback, followed by comments and reactions posted by other students

**Fig. 2:**
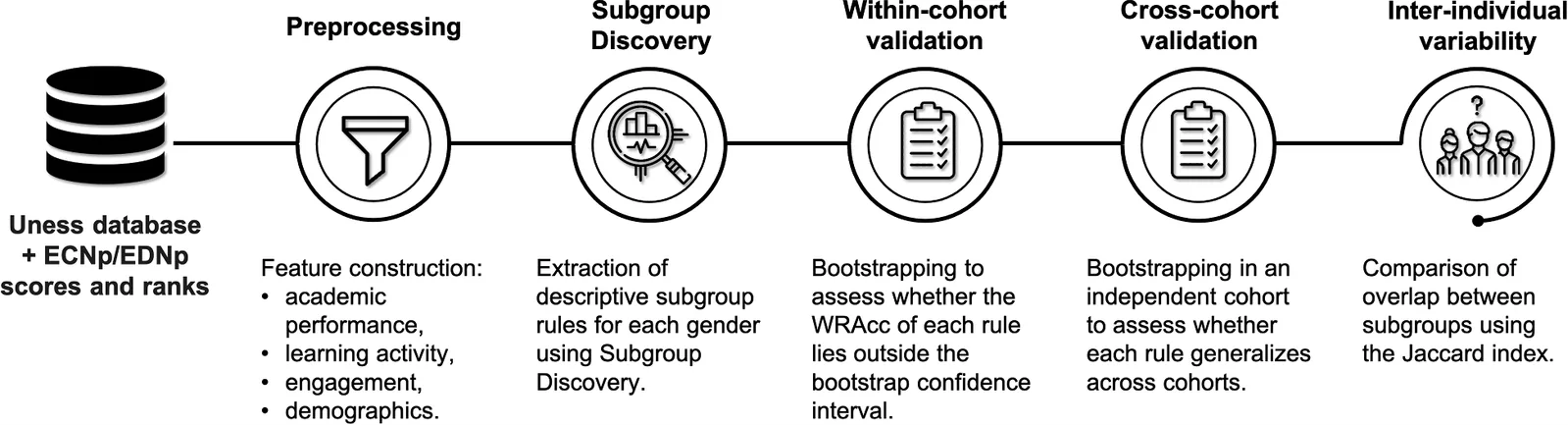
Overview of the study workflow. Starting from the UNESS database, the workflow includes data preprocessing and feature extraction, followed by *Subgroup Discovery* performed independently in both student cohorts. In the next step, the rules identified in each cohort are internally validated using bootstrapping. Subsequently, external validation is conducted by applying bootstrapping of the rules derived from one cohort to the other independent cohort. The final analyses assess interindividual variability

## Methods

### NTB platform and study

***NTB:*** We used the NTB platform provided by the National Digital University for Health and Sports (UNESS). The NTB offers medical students training resources for the national licensing examinations. For the present study, UNESS provided access to students’ activity logs from the ECN NTB. This training bank included: 4,757 PC, 158 CAR and a bank of 29,952 questions for IQS. All activities are delivered as MCQs (single-best-answer questions and multiple-answer questions).

Each format serves a different educational purpose. IQS assess factual knowledge, PC present stepwise clinical scenarios, and CAR require students to critically analyze scientific articles. Students can select from 31 specialties across all training formats, and IQS sessions allow them to choose the number of questions per session.

Upon submission, the platform provides a detailed correction report (e.g. Fig. 1) that classifies each response (e.g., “correct”, “essential”, “incorrect” or “unacceptable”). Students can comment on corrections, reply to other users, or express likes or dislikes. In addition, a private chat feature enables direct interaction between users.

Multiple-answer questions were scored using a decreasing partial-credit scheme. Each question contained four or five answer options and was graded according to the number of discrepancies between the student’s responses and the reference answers: 1 for no discrepancy, 0.5 for one discrepancy, 0.2 for two discrepancies, and 0 for three or more discrepancies. In addition, some questions included essential or unacceptable answer options. Selecting an unacceptable option or failing to select an essential option resulted in a score of 0 for the entire question, regardless of the remaining responses.

***Our study:*** Our analysis consisted of five steps: (1) preprocessing NTB raw data, (2) generating patterns via *Subgroup Discovery*, (3) validating the patterns through bootstrapping, (4) testing their generalizability on an independent cohort (either 2022 or 2023), and (5) examining overlaps among rules to study inter-individual variability. This workflow is illustrated in Fig. 2.

**Table 1:** Set of extracted features for the analysis of demographic data as well as online behaviors and performances. Variables containing [exercise type] or [specialty] represent families of derived variables generated for each exercise type and medical specialty. The complete list is provided in Supplementary Table S6

Extracted features			
Learning activity	Engagement	Performance	Demographic
NbDays	NbComments	AcademicPerformance	University
Density	NbDislikesGiven	AcademicRank	Gender
Frequency	NbLikesGiven	CorrectChoice	
TimeOnTask	CommentsSize	IncorrectChoice	
nb_[exercise type]_unique	AvgDislikesReceived	EssentialChoice	
nb_[exercise type]_all	AvgLikesReceived	UnacceptableChoice	
[specialty]_nb_[exercise type]	AvgAnswer	EvolutionScore	
	NbMessages	AbandonedQuestions	
		avg_[exercise type]_all	
		[specialty]_avg_[exercise type]	

### Data preprocessing

We analyzed anonymized data from sixth-year medical students in the 2022 and 2023 cohorts who participated in all preparatory assessments (called ECNp or EDNp) and interacted with the NTB platform. This resulted in a dataset of 6,553 students (2022) and 6,279 students (2023) across 32 French medical universities.

The ECNp/EDNp results have been added to the NTB platform data (AcademicRank and AcademicPerformance variables). A total of 204 attributes were extracted and grouped into four categories: learning activity, engagement, performance, and demographics (Table 1; complete descriptions are provided in Table S6 in Supplementary File). Learning activity attributes describe training frequency and duration, engagement attributes track student interactions (e.g., comments, likes), performance attributes capture quiz results by specialty and exercise type, and demographic attributes include university and gender.

Gender was determined using official UNESS records whenever available. Otherwise, gender was inferred from first names using the *Gender-Guesser* Python library, retaining only classifications returned as “male” or “female” (ambiguous and unisex classifications were excluded). For compound first names, each component was evaluated separately, and gender was assigned only if all components yielded the same classification. The first names left unassigned by *Gender-Guesser* were classified using *ChatGPT 3.5* to create male and female first-name lists. Names that could not be classified by any method were coded as missing. Further information on this method and its validation is available in Section A of the Supplementary File.

For activity-specific variables, a value of −1 indicates that the student did not participate in the corresponding activity rather than representing a missing value. Valid performance values are bounded below by 0.

### Subgroup Discovery analysis

We utilized a *Subgroup Discovery (SD)* approach, whose mathematical formulation is provided in Section B of the Supplementary File. Instead of testing predefined hypotheses, *SD* identifies interpretable subgroups that differ from the overall population with respect to a predefined target variable. Specifically, it uncovers groups of learners who share common behaviors and are enriched for a target characteristic (e.g., female) relative to the remainder of the dataset.

The extracted patterns are presented as “if-then" rules (i.e., ‘A implies B’), where A is a combination of conditions on various factors (e.g., ‘Urology_nb_CAR*=0* AND NbLikesGiven*≥ 4*’) and B is a characteristic of interest, called a target class (e.g., ‘male’). For example, the rule ‘Urology_nb_CAR*=0* AND NbLikesGiven$\ge 4 \rightarrow$ male’ indicates that men are overrepresented among students who did not complete a urology CAR and gave more than four likes. The algorithm selected the ranges or cut-off values that best differentiate the target class by optimizing a quality measure named *WRAcc* [29].

*WRAcc* quantifies the difference between the prevalence of the target class within a subgroup and its prevalence in the overall dataset, weighted by the prevalence of the target class in the dataset. High-*WRAcc* rules therefore identify subgroups that are both distinct from the overall population and sufficiently large to represent meaningful behavioral patterns.

We used the Python library *pysubgroup* [30] to extract behavioral patterns within each class. The algorithm explores the search space using a depth-first search strategy and returns the *K* highest-ranked patterns according to the *WRAcc* quality measure. Bootstrapping (described in Section B of the Supplementary File) was then used to assess their statistical significance [31]. All patterns presented in the following section were statistically significant. Patterns validated in one cohort were subsequently evaluated in the second cohort to assess their generalizability.

Because this study investigated gender-specific differences in learning behaviors, the target classes (B) were gender (female and male). The descriptive factors (A) considered in the analysis comprise all the variables described in Table 1.The analysis therefore aimed to identify the behavioral and performance factors that best characterize male and female students.

### Study of inter individual variability

We present results using a maximum search depth of 1, because multidimensional patterns were harder to interpret. Consequently, each subgroup description contains a single descriptive attribute. To analyze inter-individual variability, we compared subgroups using the *Jaccard Index (IoU)*, which measures the proportion of individuals shared by two subgroups. The *IoU* is defined in Section D of Supplementary File. An *IoU* of 1 indicates complete overlap, meaning that two rules identify the same individuals, whereas a value of 0 signifies completely distinct subgroups.

**Fig. 3:**
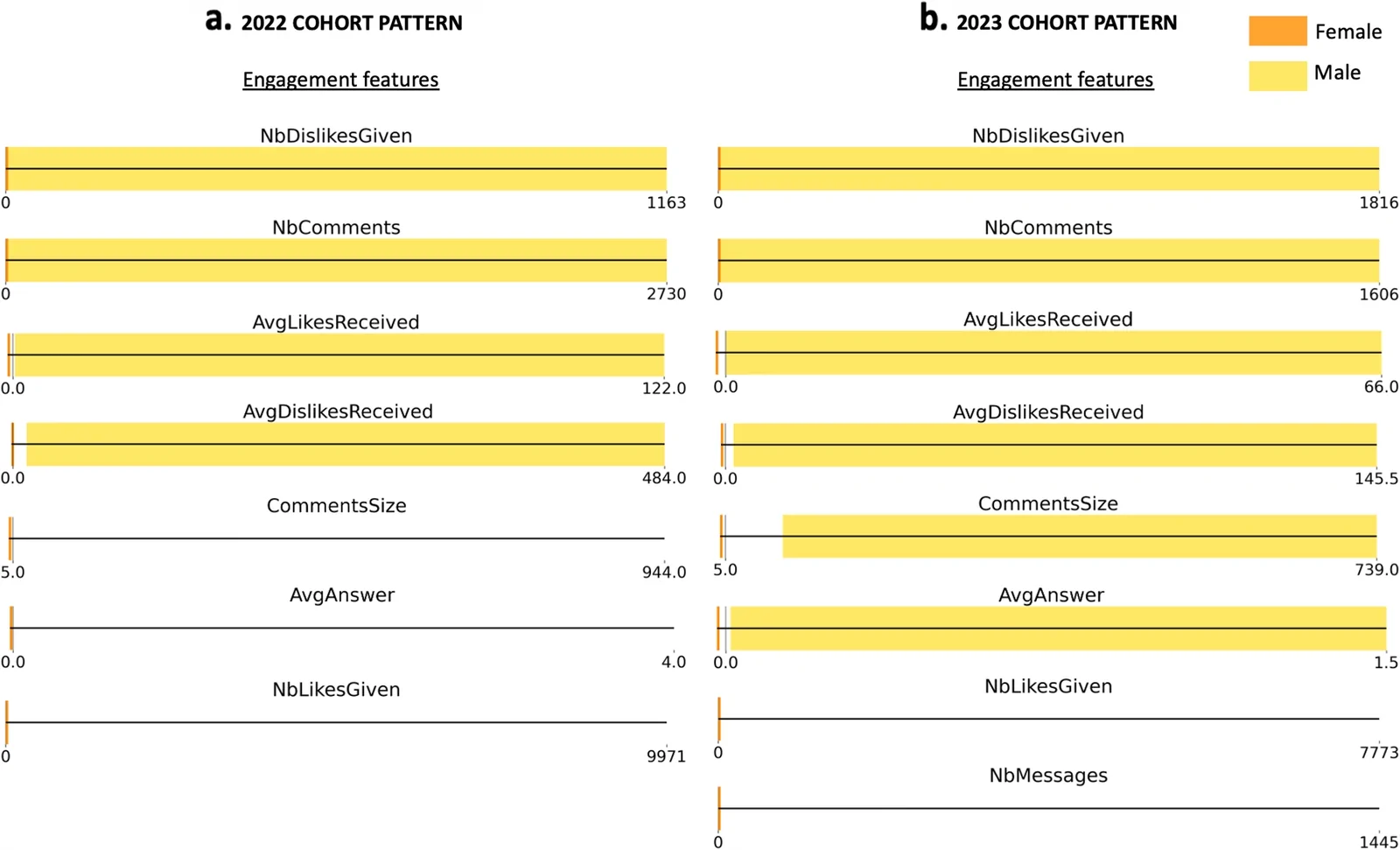
Visualization of the engagement-related subgroup patterns identified in the 2022 (**a**) and 2023 (**b**) student cohorts. Reported values correspond to the observed minimum and maximum for each attribute. Colored bars represent the value ranges defining each rule for male and female students. Black horizontal lines indicate the full range of observed values for each attribute, from the minimum (or -1, when applicable) to the maximum. A grey vertical line indicates the minimum observed value. When a line appears below zero, it corresponds to the value *-1*, indicating the absence of training for that attribute

**Fig. 4:**
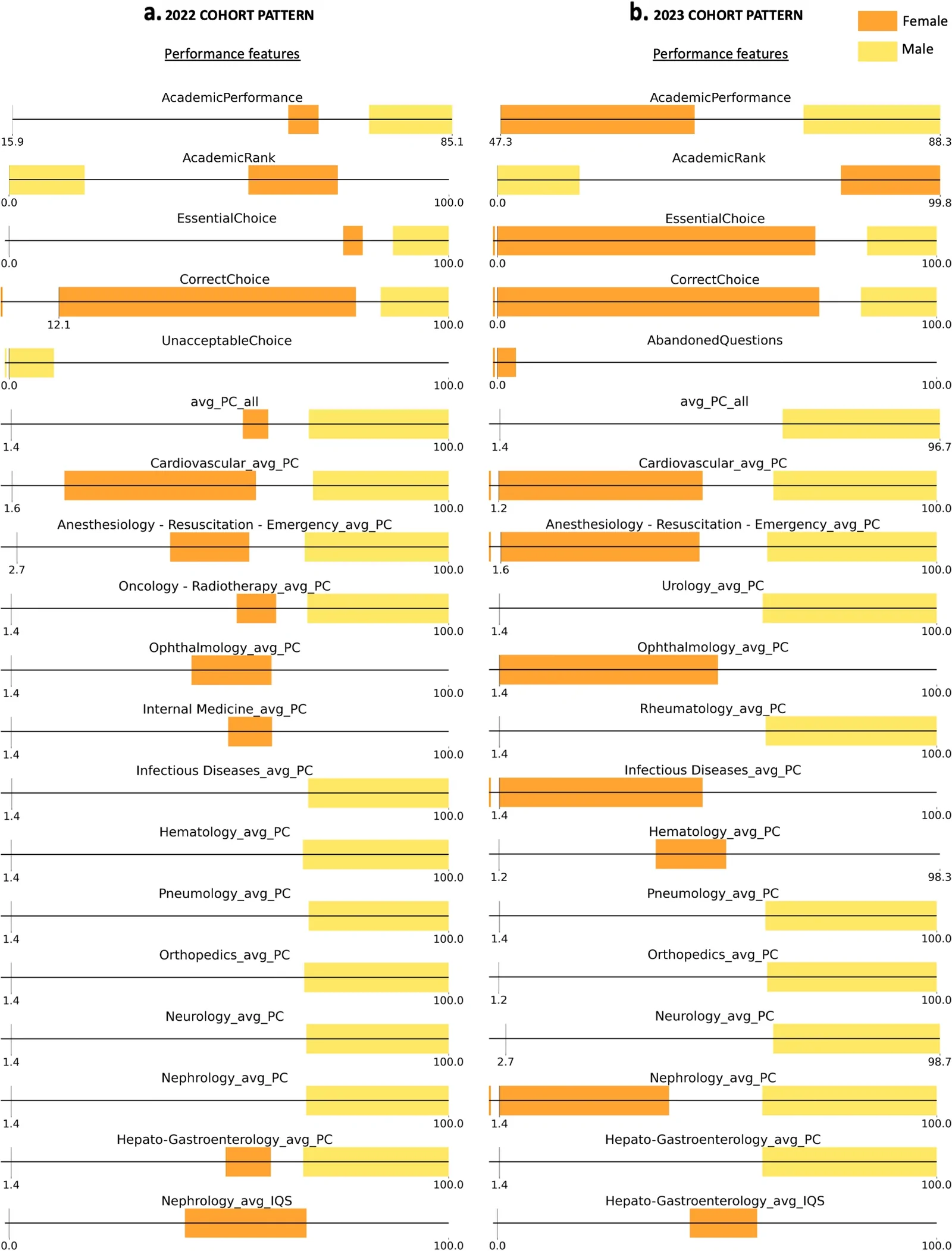
Visualization of the performance-related subgroup patterns identified in the 2022 (**a**) and 2023 (**b**) student cohorts. Reported values correspond to the observed minimum and maximum for each attribute. Colored bars represent the value ranges defining each rule for male and female students. Black horizontal lines indicate the full range of observed values for each attribute, from the minimum (or -1, when applicable) to the maximum. A grey vertical line indicates the minimum observed value. When a line appears below zero, it corresponds to the value *-1*, indicating the absence of training for that attribute

**Fig. 5:**
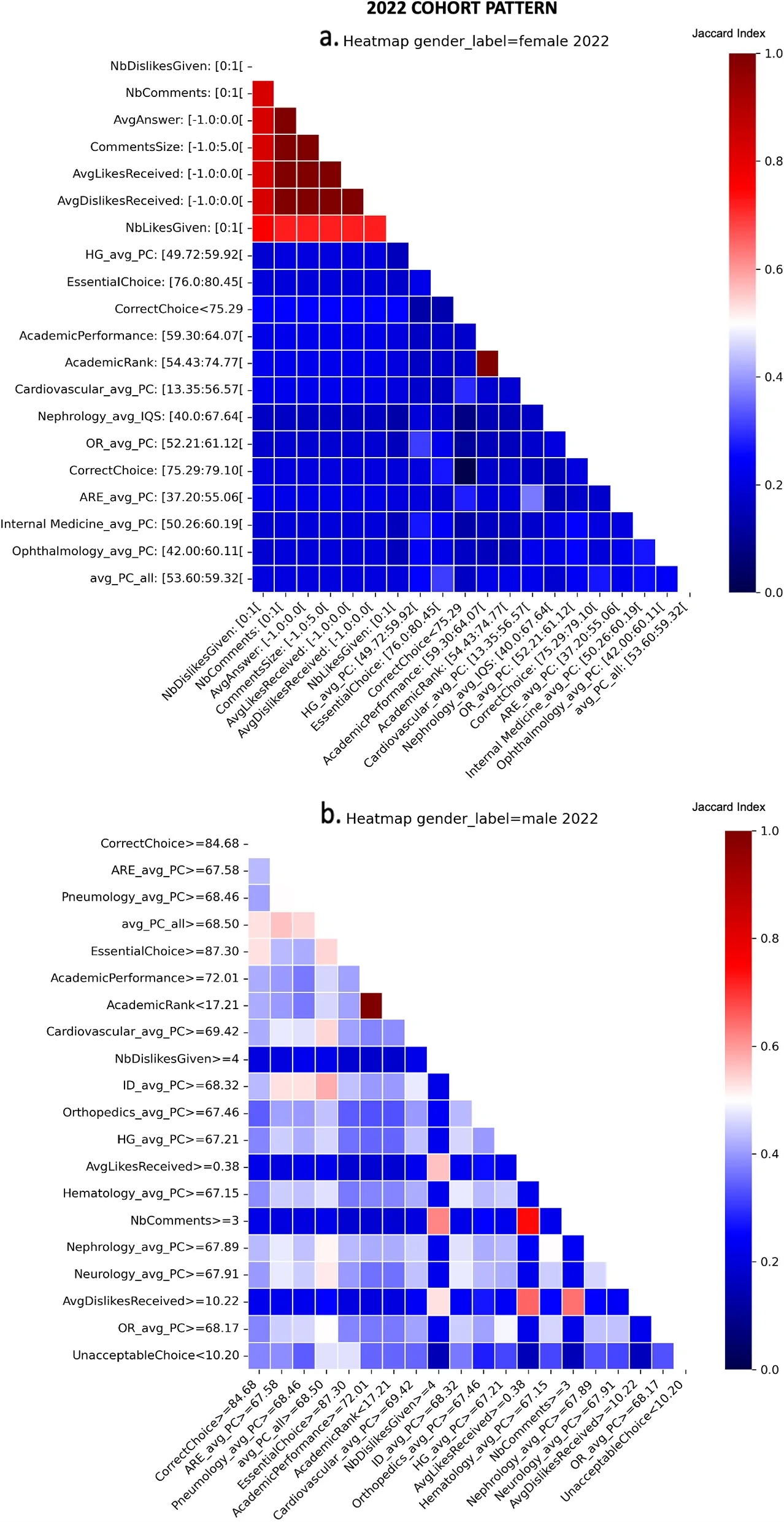
Pairwise *Jaccard Index* between subgroup patterns identified within the **2022 cohort** for female (**a**) and male (**b**) students. Each cell represents the overlap between two patterns. A *Jaccard Index* close to 0 (dark blue) indicates that the corresponding patterns are disjoint, whereas a *Jaccard Index* close to 1 (dark red) indicates that they strongly overlap

**Fig. 6:**
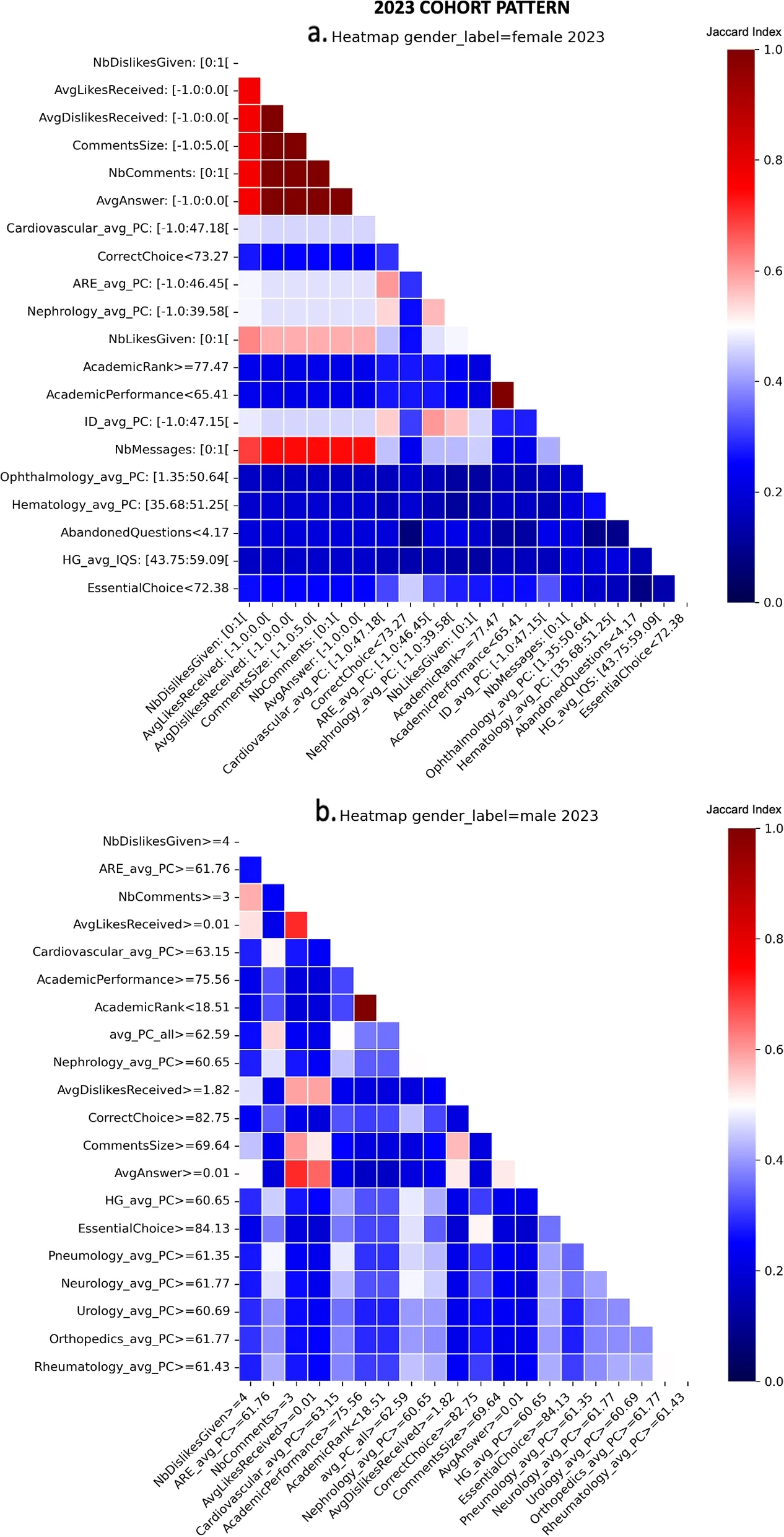
Pairwise *Jaccard Index* between subgroup patterns identified within the **2023 cohort** for female (**a**) and male (**b**) students. Each cell represents the overlap between two patterns. A *Jaccard Index* close to 0 (dark blue) indicates that the corresponding patterns are disjoint, whereas a *Jaccard Index* close to 1 (dark red) indicates that they strongly overlap

## Results

This section identifies factors associated with gender to determine whether gender inequalities exist and to characterize their nature. We extracted subgroups of individuals for each gender target class. For each cohort and gender, the twenty most relevant patterns were retained. The complete set of extracted patterns, together with their *WRAcc* values, support, and the results of both bootstrap validations, is provided in Tables S1–S4 of the Supplementary File.

The validated patterns are illustrated in Figs. 3 and 4, where each rule is represented by a rectangle.

As an illustrative example, we focus on the interpretation of the Correct Choice patterns in the 2022 cohort. The proportion of correct choices selected ranged from 12.1% to 100%, yielding two significant patterns. The yellow pattern (“$CorrectChoice \ge 84.68 \rightarrow male$") indicates that participants who selected at least 84.68% of the correct choices were overrepresented among males relative to the overall gender distribution of the dataset. The orange pattern (*CorrectChoice < 73.27 → female*) indicates that participants who selected fewer than 73.27% of the correct choices, including those who were not presented with any questions containing correct choices (−1), were overrepresented among females, whereas males were underrepresented in this group. No significant gender-specific pattern was identified for participants with a Correct Choice rate between 73.27% and 84.68%.

Most rules (68.75%) relate to performance, with the remainder involving engagement. No validated patterns were identified for learning activity or university affiliation. Bootstrap validation confirmed all patterns within their respective cohorts, and most also generalized across cohorts, except a few female-specific performance rules. Descriptive statistics by gender for the attributes most frequently identified in the subgroup rules are provided in Supplementary Table S5.

Figure 3 illustrates the engagement rules: students who did not engage with the platform were predominantly female, whereas those who engaged were predominantly male. The Jaccard index analysis (Figs. 5, 6, and Supplementary Fig. S2) showed that engagement-related rules consistently exhibited the highest pairwise overlap within each cohort. Mean Jaccard indices reached 0.62 and 0.57 for men in 2022 and 2023, respectively, compared with 0.79 and 0.88 for women. This suggests that engagement rules predominantly capture the same subsets of students, particularly among women.

Figure 4 presents the performance rules. Across both cohorts, individuals with better results on national preparatory exams and online training sessions —particularly in progressive cases for nephrology, cardiovascular medicine, and anesthesiology-resuscitation-emergency (ARE)— were predominantly men. Performance-related rules exhibit a more heterogeneous overlap than engagement rules. Among male students, the mean pairwise Jaccard index between performance rules was 0.43 in the 2022 cohort and 0.39 in the 2023 cohort, indicating that several performance patterns describe partially overlapping student populations. In contrast, female performance-related rules show lower overlap, with mean pairwise Jaccard indices of 0.21 in 2022 and 0.25 in 2023, suggesting that the identified performance patterns describe more distinct subgroups.

Pairwise overlap between engagement-related and performance-related rules remained low across all cohorts, with mean Jaccard indices ranging from 0.21 to 0.29. This limited overlap suggests that engagement- and performance-related rules captured largely distinct subsets of students. Among women in the 2023 cohort, engagement–performance rule pairs exhibited greater variability in overlap (Jaccard index range: 0.12–0.49; Supplementary Fig. S2) than those in the other cohorts, although the average overlap remained modest (mean Jaccard index = 0.29).

Regarding performance-related patterns, the vast majority involved PC (13 patterns per cohort), whereas only one pattern per cohort involved IQS, and none were related to CAR. The remaining patterns were associated with overall performance on the mock national examinations (ECNp/EDNp) and the severity of the selected choices (essential and correct). Among women, seven performance attributes (AcademicPerformance, AcademicRank, EssentialChoice, CorrectChoice, Cardiovascular_avg_PC, ARE_avg_PC, and Ophthalmology_avg_PC) were identified in both cohorts. Comparison of the corresponding patterns indicates average performance in 2022 but either no training or very low scores in 2023, indicating a shift in the performance profile of female-associated patterns between the two cohorts. Because data prior to 2022 were unavailable, we could not determine whether this patter predated our study.

**Fig. 7:**
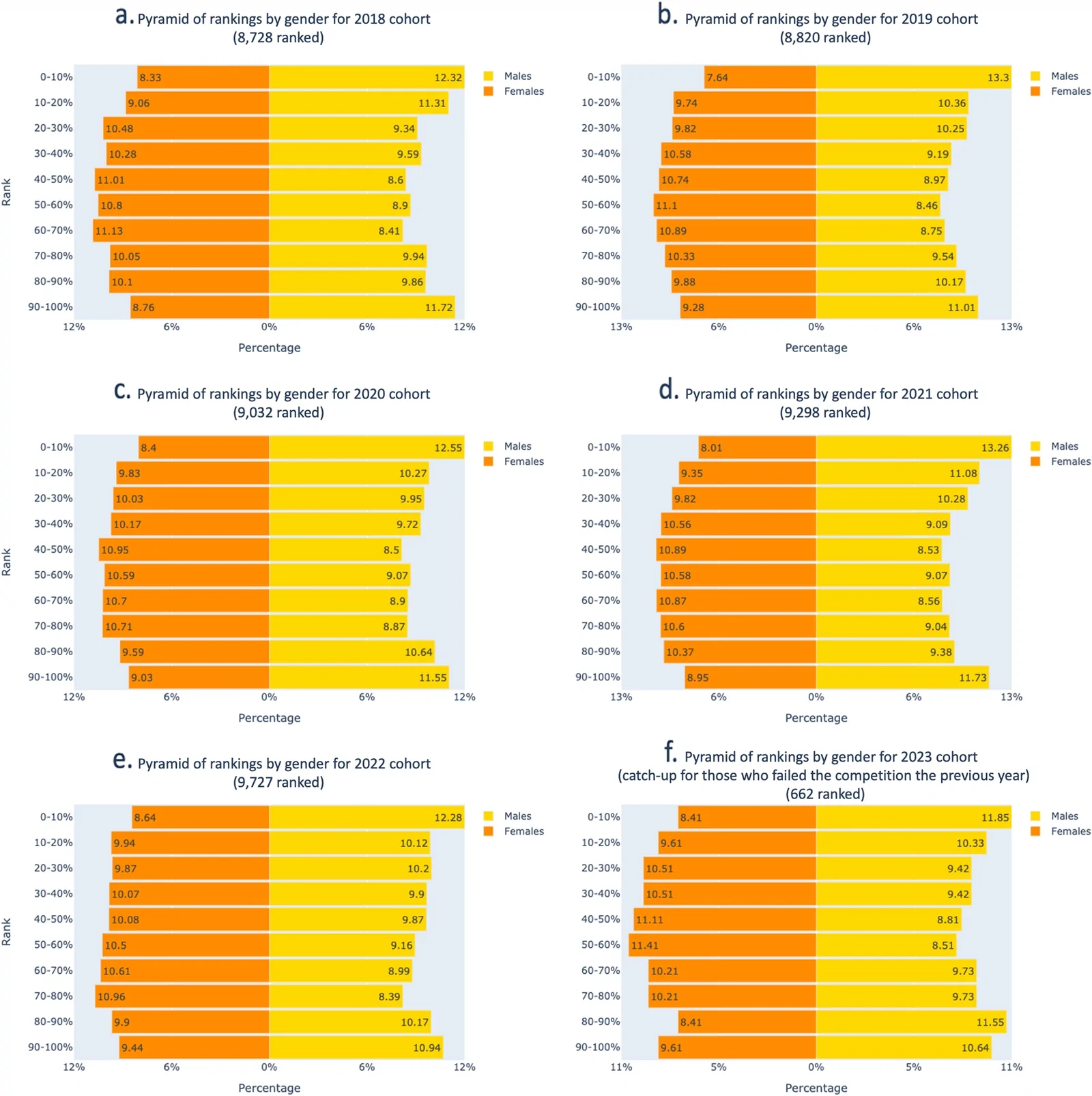
Distribution of male and female students across 10-percentile intervals of the official ECNi ranking for each cohort from 2018 to 2023. Each horizontal bar represents the percentage of students of a given gender within a ranking interval

To further interpret these findings, we examined the official ECNi ranking distributions from 2018 to 2023 (Fig. 7). These data provide additional context to assess whether the gender differences observed in our 2022 and 2023 cohorts were consistent with previous national examinations. Official ECNi results indicate stable national patterns: women are more frequently represented in the middle of the ranking, whereas men are overrepresented at both the top 10% and the bottom 10%. This indicates that the gender differences identified in the 2022 cohort are consistent with patterns observed in previous national examinations. Unfortunately, official EDN data are not publicly available, precluding comparisons beyond 2022. Consequently, we could not determine whether the trend observed in the 2023 cohort reflects a broader change.

For women in 2022, the 13 performance patterns largely described distinct subgroups. Some were characterized by lower engagement, whereas others exhibited weaknesses in one or more specialties. For the 2023 cohort, performance patterns involving infectious diseases (ID), cardiovascular medicine, ARE and nephrology identified overlapping populations, with a notable 42–49% overlap with engagement patterns. Other performance patterns identified to distinct populations. These findings suggest that some women combined low engagement with limited training or poor performance in ID, ARE, cardiovascular, and nephrology, whereas others exhibited weaknesses restricted to specific specialties.

## Discussion

Our results highlight marked gender disparities in engagement and performance among students in French medical schools. In particular, the differences observed in overall performance appear to be primarily linked to “progressive cases” and response patterns suggesting that some female students are more likely to omit correct answer choices.

### Performance disparities

These performance disparities may begin early in post-secondary education, as official statistics from the French Ministry of Education [32–35] show that women accounted for up to 70% of first-year students in the health-related programs; but only 64–66% of students in the the following year. Similar disparities have also been reported in medical education in the United States, where men experience fewer academic difficulties whereas women perform better in practical clinical examinations [36].

#### Assessment design

Our results are based exclusively on MCQs assessments and show lower performance among female students. Previous studies suggest that assessment format itself may contribute to such differences. Girls outperform boys in assessments involving written work, oral presentations, and classroom participation, whereas boys perform better on standardized MCQs examinations [37]. Likewise, large-scale educational studies consistently report a relative male advantage on MCQs and a female advantage on constructed-response items [38, 39]. These findings support the hypothesis that assessment format contributes to gender differences in academic performance.

Several mechanisms have been proposed to explain these differences. One relates to response strategies under uncertainty. Baldiga [40] and Pekkarinen [41] showed that, when incorrect answers are penalized, female students leave more questions unanswered despite comparable knowledge levels. Saygin et al. [42] further showed that this behaviour is partly explained by self-assessment: students who perceive themselves as more competent are more likely to answer uncertain questions rather than leave them blank. At equivalent academic performance, men rated themselves as more competent than women in mathematics, science, and social sciences, suggesting that self-confidence contributes to gender differences in response behaviour. This mechanism is consistent with our observation that female students more frequently omitted correct answer choices. Although the ECNi/EDN does not use negative marking, its scoring system introduces an implicit cost of error through progressively reduced partial credit and non-compensatory rules, whereby selecting an unacceptable answer or omitting an essential one results in a score of zero for the entire question. These scoring rules may similarly encourage more conservative response strategies under uncertainty.

Psychometric studies further suggest that the characteristics of individual MCQ items may also contribute to gender differences. In physics assessments, items favouring male students more frequently involved visual reasoning, whereas items favouring female students relied more often on straightforward numerical calculations [43]. Medical examinations include both visual interpretation (e.g., radiology, dermatology, anatomy) and numerical calculations (e.g., drug dosage or clinical scores). Future item-level analyses could therefore determine whether specific question characteristics contribute to the disparities observed in our study.

Nearly all studies discussed above concern single-best-answer MCQs, whereas our study involved multiple-response MCQs. In this format, each option represents a separate decision under uncertainty, and scoring combines partial credit with non-compensatory rules for essential and unacceptable answers. If response strategies already differ between genders in conventional MCQs, these differences may be amplified in multiple-response examinations. To our knowledge, this hypothesis has not yet been investigated.

#### Assessment context

The high-stakes nature of the French national medical examinations may also contribute to the observed disparities. In other academic fields, examination pressure has been shown to affect female students more strongly than male students, both in class [44] and online settings [45]. Interestingly, Montolio et al. [44] further reported that this effect was most pronounced among high-performing students. This finding may partly explain the stable ECNi ranking distributions observed between 2018 and 2022, where women were consistently overrepresented in the middle of the ranking but underrepresented among the highest-ranked candidates.

Time constraints represent another potential mechanism. The ECNi and EDN consist of three-hour examinations including approximately 180 to 220 questions. Stoevenbelt et al. [46] showed that under strict time limits, women tend to complete fewer items than men, whereas these differences decrease when time constraints are relaxed. Their results suggest that part of the observed gender gap reflects differences in test completion rather than differences in underlying ability.

The role of stereotype threat remains less clear. Although Stoevenbelt et al. [46] found no measurable effect, Davies et al. [47] reported that stereotype threat mainly affected questions requiring intuitive reasoning or comparison rather than procedural problem solving. Its impact may therefore depend less on examination difficulty than on the cognitive processes required by individual questions.

#### Clinical reasoning

The mechanisms discussed above may contribute to the overall performance differences observed between men and women. However, they would be expected to affect all MCQ-based assessments and therefore do not explain why the observed disparities were largely confined to Progressive Cases. Unlike IQS, which primarily assess factual knowledge and problem solving, or CAR, which evaluates evidence appraisal skills, PCs are specifically designed to assess clinical reasoning in a dynamic context. Candidates must repeatedly generate, revise, and prioritize diagnostic hypotheses as new clinical information becomes available, while committing to successive management decisions under uncertainty.

Current evidence does not support a male advantage in clinical reasoning itself. Groves et al. [48] reported higher scores among female students on Clinical Reasoning Problems, which assess the integration and weighting of clinical information through constructed responses. Likewise, workplace-based assessments showed that female students outperformed men across several domains of clinical performance despite similar scores on standardized MCQ examinations [49]. Using virtual patient simulations, Hege et al. [50] further observed that female students generated more differential diagnoses, requested more diagnostic tests, and produced more comprehensive case summaries, without achieving higher diagnostic accuracy [50].

Together, these studies suggest differences in reasoning style rather than diagnostic competence. Female students appear to adopt a more comprehensive and analytically detailed approach, whereas diagnostic accuracy remains comparable between genders. One possible explanation is that such reasoning strategies, although potentially advantageous in authentic clinical settings, may be less well suited to the PC format, which combines repeated multiple-choice decisions with evolving clinical uncertainty. Success in this format may depend not only on generating multiple diagnostic hypotheses but also on efficiently discarding unlikely alternatives and committing to successive decisions as new information emerges. Consequently, PCs may favour decision-making strategies characterized by lower decision thresholds, greater confidence calibration, and earlier commitment to diagnostic hypotheses. This hypothesis could explain why the gender difference was most pronounced for PCs, weaker for IQS, and absent for CAR.

#### Specialty-specific performance

Beyond the overall performance differences, *subgroup discovery* repeatedly identified the same medical specialties among the strongest discriminative attributes. This raises the question of why gender differences were concentrated in these specialties rather than being uniformly distributed across the curriculum.

Evidence regarding gender differences in specialty-specific performance remains scarce. Previous studies consistently reported higher examination performance among female students in obstetrics and gynecology, psychiatry, and pediatrics [51–54], whereas little evidence is available for the specialties identified in our study.

Previous work suggests that specialty-related performance differences may partly reflect differences in clinical exposure, confidence, and career interests. Female medical students report less procedural exposure and lower confidence during surgical clerkships despite similar initial interest in surgery [55]. They also tend to favor specialties focused on the patient relationship (pediatrics, obstetrics and gynecology, and psychiatry), whereas male students more frequently choose technical or surgically oriented disciplines (orthopedics, urology, cardiothoracic surgery, and emergency medicine) [56–59].

Our findings therefore suggest several non-exclusive hypotheses. Specialty-specific performance differences may reflect differences in clinical exposure, confidence, or career interests. They may also arise because some specialties rely more heavily on assessment characteristics discussed above, such as clinical reasoning under uncertainty, visual interpretation, or other specialty-specific question features. Distinguishing between these explanations will require future studies combining item-level analyses with measures of students’ educational experiences and specialty preferences.

### Engagement disparities

Previous studies generally report only modest gender differences in learning behaviours [60, 61], although female medical students tend to make greater use of self-regulated learning strategies such as planning, help-seeking, and self-evaluation [62–65]. These findings appear inconsistent with the lower engagement observed among women on the NTB platform. However, the indicators analysed in our study (posting comments and receiving reactions) capture a specific form of online social participation rather than overall learning engagement or study habits. Consequently, lower visible participation on the NTB platform should not be interpreted as lower engagement in learning, but rather as lower engagement in one specific form of online social interaction.

The medical e-learning literature provides limited insight into these behaviours. Most studies have relied on self-reported measures of satisfaction, acceptance, or perceived usefulness rather than objective behavioural data, and their findings remain inconsistent, with some reporting greater acceptance among male students [66, 67] and others more favourable perceptions among female students [68].

Outside medical education, studies conducted in MOOCs and other online learning environments indicate that gender differences in online participation depend strongly on course discipline, design, and learning environment [69, 70]. Moreover, previous work has focused on forum participation, while other forms of online interaction, such as likes, dislikes, or voting behaviours, have received little attention and have rarely been analysed from a gender perspective [71]. Our study extends this literature by showing that male students not only contributed more comments but also received more reactions, suggesting greater visibility and interaction within the online learning community.

Finally, psychosocial factors may also contribute to these differences. Female medical students report higher rates of depression, anxiety, and psychological distress than their male counterparts [72]. These conditions have been associated with reduced academic engagement and participation in both online [73] and face-to-face learning environments [74], and may partly explain why female students engaged less frequently in visible online interactions. Future studies integrating psychological assessments could help clarify the extent to which these factors contribute to the gender-specific online engagement patterns observed in our study.

### Conclusion on the ECNi/EDN format

These observations underscore the need for targeted strategies and further research to promote equitable participation and career opportunities in medical education. The transition from the ECNi to the EDN, through the introduction of assessment formats beyond conventional MCQs—including short open-ended response questions and image-based pointing questions—together with the implementation of OSCE, represents a promising step toward a broader assessment of clinical competencies. However, diversifying assessment formats alone may not be sufficient. Future research should also investigate the influence of scoring rules, time constraints, response strategies under uncertainty, and the cognitive characteristics of individual questions on gender-specific performance. Item-level psychometric analyses may help identify assessment formats or scoring mechanisms that unintentionally disadvantage particular groups of students while preserving the validity of national examinations. Although the impact of the EDN reforms remains to be evaluated, such evidence could guide the design of future assessments that evaluate a wider range of competencies while minimizing unintended sources of inequality.

Finally, our study was descriptive and therefore cannot distinguish between the different mechanisms proposed to explain the observed gender disparities. Rather than providing causal explanations, our findings, together with the existing literature, generate testable hypotheses that future studies should investigate using item-level analyses, response-process data, psychological measures, and comparisons across assessment formats.

### Methodological insights and future directions

Rather than testing predefined hypotheses, our approach used *Subgroup Discovery* to automatically identify interpretable behavioural patterns associated with gender. The extracted rules provide explicit, reproducible descriptions of learner subgroups that generate testable hypotheses for future research. More broadly, the same framework could be applied to investigate other learner characteristics or educational questions beyond gender.

Several limitations should nevertheless be acknowledged. First, gender was represented as a binary variable and therefore does not capture the full diversity of gender identities. Second, although gender was obtained from official records whenever possible, it had to be inferred from first names for part of the 2023 cohort, introducing a potential source of misclassification. The consistency of the subgroup patterns across cohorts nevertheless suggests that this limitation had a limited impact on the principal findings. Third, our analyses focused exclusively on gender, whereas other characteristics, such as age [36, 75–77] and socioeconomic background [75, 76, 78, 79], are also known to influence medical learning.

The present study limited the rules in terms of quality, number, and depth. Future work could generate more rules and leverage reinforcement learning to refine rule selection, prioritizing the most meaningful descriptive rules based on user preferences. Alternatively, a top-down approach could validate expert- or student-defined hypotheses [80], ensuring representativeness and enhancing the relevance of findings for medical education. Additional validation strategies could also be investigated to quantify rule stability under repeated data perturbations and further assess the robustness of discovered subgroup patterns.

Finally, the recent introduction of the EDN assessment format together with OSCE provides an opportunity to evaluate whether the gender disparities observed in this study persist under a substantially different assessment framework. Since completion of the present study, the NTB platform has also introduced dedicated EDN learning resources that were not available during our analyses. Future studies based on these new resources could determine whether changes in assessment design are accompanied by changes in online learning behaviours, and whether these reforms contribute to reducing gender disparities in medical education.

## Conclusion

This study reveals notable gender disparities in both online engagement and performance among French medical students through an original application of *Subgroup Discovery*. Those who are most engaged with the interactive features of the platform and who obtain better results in exams, particularly in Progressive Cases, are generally men. In contrast, female students tend to either disengage from the platform or exhibit average to lower performance in certain medical specialties with a greater tendency to omit correct answer choices.

Together with the existing literature, these findings generate several hypotheses regarding the mechanisms underlying these disparities. Assessment design, examination context, response strategies under uncertainty, and specialty-specific assessment characteristics may all contribute to the observed patterns. Future studies combining item-level analyses, psychological measures, and behavioural data will be needed to disentangle these mechanisms.

Beyond the specific findings on gender, this study illustrates how *Subgroup Discovery* can generate interpretable behavioural patterns and hypothesis-generating models from large-scale educational data. Such approaches may help identify learner profiles that remain difficult to detect using conventional hypothesis-driven analyses and thereby support more targeted educational interventions.

Finally, the recent introduction of the EDN, with more diverse assessment formats and the addition of OSCE, provides a valuable opportunity to determine whether changes in assessment design reduce the gender disparities observed in this study. Evaluating these reforms, together with future analyses of online learning behaviours, may contribute to the development of fairer assessment practices and more equitable learning environments for future physicians.

## Supplementary Information

Supplementary Material 1.

## Data Availability

Data on official statistics on gender inequality in medicine in France are available here: - 2021 : https://www.egalite-femmes-hommes.gouv.fr/sites/efh/files/migration/2021/12/Chiffres-cles-Egalite-femmes-hommes-2021.pdf -2022: https://www.enseignementsup-recherche.gouv.fr/sites/default/files/2022-03/esri---vers-l-galit-femmes-hommes-chiffres-cl-s---2022-17273.pdf - 2023: https://www.enseignementsup-recherche.gouv.fr/sites/default/files/2023-04/esr---vers-l-galit-femmes---hommes-chiffres-cl-s-2023-27332.pdf - 2024: https://www.enseignementsup-recherche.gouv.fr/sites/default/files/2024-03/vers-l-galit-femmes-hommes-chiffres-cl-s-2024-32097.pdf The platform data analyzed in this article were provided by O.P. and are available upon reasonable request.

## Abbreviations

ARE: Anesthesiology-Resuscitation-Emergency
CAR: Critical Article Readings
DPO: Data Protection Officer
ECNi: computerized National Ranking Examination
ECNp: preparatory Computerized National Ranking Examination
EDN: National Digital Examinations
EDNp: preparatory National Digital Examinations
HG: Hepato-Gastroenterology
ID: Infectious diseases
IoU: Jaccard Index
IQS: Isolated Question Sequences
KFP: Key Feature Problem
MCQ: Multiple Choice Questions
NTB: National Training Bank
OR: Oncology-Radiotherapy
OSCE: Objective Structured Clinical Examinations
PC: Progressive Cases
SD: Subgroup Discovery
UNESS: National Digital University for Health and Sports
WRAcc: Weighted Relative Accuracy
