# Supplementary material for: A study of gender-specific differences in online platform in medical education
Source: BMC Med Educ. 2026 Jul 25;26:1686. doi: 10.1186/s12909-026-10010-6 (PMC13685372; doi:10.1186/s12909-026-10010-6)
Supplement: Supplementary file 1 [file 12909_2026_10010_MOESM1_ESM.pdf]

Supplementary file of the paper: A Study of Gender-Specific Differences in Online Platform in Medical Education

Maëlle Moranges[1*], Noha Ibrahim[1], Sihem Amer-Yahia[1], Olivier Palombi[1]

[1]Computer Science Laboratory of Grenoble, CNRS, Université Grenoble Alpes, Saint-Martin-d'Hères, 38400, France.

*Corresponding author(s). E-mail(s): maelle.moranges@inria.fr;
Contributing authors: noha.ibrahim@univ-grenoble-alpes.fr;
sihem.amer-yahia@univ-grenoble-alpes.fr;
olivier.palombi@univ-grenoble-alpes.fr;

Keywords: Undergraduate medicine, Online learning, Student engagement, Assessment and performance, Educational equity, Gender differences, Subgroup Discovery, Pattern Mining, Artificial intelligence

List of Figures

S1 Example of bootstrap validation and cross-cohort validation of subgroup patterns. The curve represents the bootstrap distribution of *WRAcc* values obtained from 10,000 random groups with the same support as the evaluated pattern. The green shaded area corresponds to the 95% confidence interval, and the vertical line indicates the *WRAcc* of the evaluated pattern. A pattern is considered validated when its *WRAcc* exceeds the upper bound of the confidence interval. Panels (a) and (c) show the validation of the first- and twentieth-ranked female patterns identified in the 2022 cohort. Both patterns are validated in their original cohort. Panels (b) and (d) show the validation of these same patterns in the 2023 cohort to assess their generalizability. The first pattern remains validated across cohorts, whereas the twentieth pattern is not validated because its *WRAcc* falls within the confidence interval. 6
S2 Distribution of pairwise *Jaccard* indices according to rule category and cohort. Boxplots show the distribution of pairwise *Jaccard* indices between subgroups identified by engagement-related rules (engagement–engagement), performance-related rules (performance–performance), and engagement- versus performance-related rules (engagement–performance). For each rule category, distributions are presented separately for the four study populations (men 2022, men 2023, women 2022, and women 2023). The central line represents the median, the box spans the interquartile range (IQR), whiskers extend to 1.5 $\times$ IQR, and points beyond the whiskers indicate outliers. Higher *Jaccard* values indicate greater overlap between the sets of students identified by two rules. 11

List of Tables

S1 Extracted patterns by the *Subgroup Discovery* algorithm for the target "**female from the 2022 cohort**", indexed by their quality measure (from highest to lowest). The table highlights the rule description (column 2), its *WRAcc* value (column 3), its support (proportion of students covered by the rule; column 4), whether bootstrap validation supports this rule on the current cohort (column 5), and whether bootstrap validation supports this rule on the second cohort (column 6). 7
S2 Extracted patterns by the *Subgroup Discovery* algorithm for the target "**male from the 2022 cohort**", indexed by their quality measure (from highest to lowest). The table highlights the rule description (column 2), its *WRAcc* value (column 3), its support (proportion of students covered by the rule; column 4), whether bootstrap validation supports this rule on the current cohort (column 5), and whether bootstrap validation supports this rule on the second cohort (column 6). 8

S3 Extracted patterns by the *Subgroup Discovery* algorithm for the target "**female from the 2023 cohort**", indexed by their quality measure (from highest to lowest). The table highlights the rule description (column 2), its *WRAcc* value (column 3), its support (proportion of students covered by the rule; column 4), whether bootstrap validation supports this rule on the current cohort (column 5), and whether bootstrap validation supports this rule on the second cohort (column 6). 9
S4 Extracted patterns by the *Subgroup Discovery* algorithm for the target "**Male from the 2023 cohort**", indexed by their quality measure (from highest to lowest). The table highlights the rule description (column 2), its *WRAcc* value (column 3), its support (proportion of students covered by the rule; column 4), whether bootstrap validation supports this rule on the current cohort (column 5), and whether bootstrap validation supports this rule on the second cohort (column 6). 10
S5 Descriptive statistics by gender for the main attributes identified in the *Subgroup Discovery* analysis. The table reports the mean and standard deviation of the attributes most frequently selected in subgroup rules across cohorts and genders. Means and standard deviations were computed using only valid observations (values 0). The percentage of students with a value of -1, corresponding to the absence of activity for the considered attribute, is reported separately. 12
S6 Complete list of the attributes included in the analyses, together with their descriptions. 16

A Approach to gender in this study

A.1 Definition of gender in the study

A binary categorization of sex (male/female) is generally assigned at birth based on the visible external anatomy of the newborn, known as "sex assigned at birth." This categorization assigns the child a title (Mr. or Ms.) as well as a usually gendered first name. In France, starting at the age of 16 (with the consent of a legal representative) or upon reaching adulthood, it is possible to request a court to change this sex designation and the first name in civil status records to align with the individual's gender identity. However, this modification remains within a binary framework (male or female).

In our study, UNESS relies on the title, and in its absence, on the first name of the students to determine their gender, which means that we are considering the official gender of the students.

A.2 Gender assignment procedure

The gender assignment procedure differed slightly between cohorts because official records were not available for all students.

For the 2023 cohort, gender was determined from official UNESS records for 7,973 individuals, from *Gender-Guesser* for 205 individuals, and from *ChatGPT 3.5* for 32 individuals; no individuals remained unclassified.

For the 2024 cohort, gender was determined from official UNESS records for 47 individuals, from *Gender-Guesser* for 6,117 individuals, and from *ChatGPT 3.5* for 190 individuals; no individuals remained unclassified.

A.3 Validation of the gender assignment procedure

To assess the reliability of the gender assignment procedure, we applied the same inference pipeline (*Gender-Guesser* followed by *ChatGPT*) to the 2022 cohort for which official UNESS gender records were available. Among the 7,973 students, the procedure correctly assigned gender for 98.53% of individuals. *Gender-Guesser* correctly classified 6,711 of 6,735 inferred names (99.64%), while the *ChatGPT* correctly classified 1,145 of 1,238 names (92.49%).

B Subgroup Discovery Analysis

Our preprocessing resulted in dataset D_1 for the 2022 cohort and dataset D_2 for the 2023 cohort. The combined dataset $D = \{D_1, D_2\}$ comprises a set S of n students, a set A of k descriptive attributes regarding student performance, engagement, learning activity, and demographic information. For the gender attribute, we defined a set T representing the classes of interest. By convention, we denote x_i as the ith attribute in the set of attributes X:

$S = \{s_1, ..., s_n\}$;

$A = \{a_1, ..., a_k\}$;

$T = \{'female', 'male'\}$.

The value of the attribute $a \in A$ for a student s is denoted $a(s)$. The gender class label of a student s is $gender(s)$. The set of students in the dataset D_j corresponding to a class label $t \in T$ is:

$$D_j^t = \{s \in D_j | gender(s) = t\} \tag{1}$$

We employed a *Subgroup Discovery* approach to identify a subset of students that characterizes the target class t relative to other classe ($\neg t$) for an attribute of interest (here, gender). This involves finding conditions on descriptive attributes strongly associated with the target class, formalized by a descriptive rule. A descriptive rule r, denoted as $r : d \to t$, is defined by a description d strongly associated with t. The description d is an ensemble of intervals $d = \{[x_1, y_1], [x_2, y_2], ..., [x_k, y_k]\}$, each restricting the value of a descriptive attribute. For example, the rule: '*Gerontology_nb_CAR*$\in$ $[0, 0]$ *AND NbDislikesGiven*$\in$ $[4; +\infty[\to$ *male*' (equivalently, '*Gerontology_nb_CAR* $= 0$ *AND NbDislikesGiven* $>= 4 \to$ *male*') means that Gerontology_nb_CAR equals zero and NbDislikesGiven is at least 4 imply the student is male. Unspecified attributes have ranges $]-\infty; +\infty[$.

Students whose attribute values satisfy d form the coverage of r:

$$cov(r) = \{s | \forall i = 1...k, x_i \leq a_i(s) \leq y_i\} \tag{2}$$

Our goal: We aim to discover subgroups where a target class is overrepresented while others are underrepresented.

To achieve this, we focus on subgroups that maximize a quality measure. Among various measures, we chose *Weighted Relative Accuracy (WRAcc)*, due to its ability to effectively balance coverage and reliability of the generated rule. This measure represents the difference between the probability of the target being present in the subgroup and the probability of the target being present in the entire dataset, multiplied by the probability of the target in the dataset.

$$WRAcc(j, r, t) = \frac{|cov(r)|}{|D_j|}\left(\frac{|cov(r) \wedge D_j^t|}{|cov(r)|} - \frac{|D_j^t|}{|D_j|}\right) \tag{3}$$

The objective of a *Subgroup Discovery* algorithm is to identify attribute intervals optimizing *WRAcc* for a target class. We used the Python library *pysubgroup* to extract behavioral patterns within each class. The search space was generated using `ps.create_selectors()`. This function automatically transforms each descriptive attribute into a finite set of candidate selectors. Categorical attributes were represented by equality selectors, whereas numerical attributes were discretized into five interval selectors using the default selector generation procedure implemented in *pysubgroup*. This procedure derives candidate intervals from the empirical distribution of each variable while adapting interval boundaries when repeated quantiles occur, thereby avoiding degenerate intervals. The resulting search space contained $1,022$ to $1,025$ candidate selectors depending on the cohort. We used the `SimpleDFS` algorithm, which performs a depth-first exploration of this finite search space while optimizing *WRAcc*. To limit complexity and improve interpretability, the maximum rule depth was set to 1. Consequently, each retained rule was defined by a single selector rather than by a conjunction of several conditions. This choice was motivated by two considerations. First, numerous statistically significant single-attribute rules were identified, providing simple and directly interpretable descriptions of gender-specific behaviors. Second, potential relationships between these rules were subsequently investigated through Jaccard-index analyses, allowing us to determine whether distinct behavioral and performance patterns characterized the same or different student populations.

To ensure result significance, each pattern undergoes bootstrap validation. We generate 10,000 random groups matching the pattern's support and compare their *WRAcc* to the original pattern. A pattern is considered validated if its *WRAcc* falls outside the 95% confidence interval of the *WRAcc* values derived from the random distribution. To implement this validation, we calculate the interval between the 100α-th and $100(1-\alpha)$ percentiles of the bootstrap distribution, corresponding to $\alpha = 0.025$. Only patterns with a *WRAcc* equal or greater than the 97.5th percentile are retained, while those that do not meet this threshold are rejected. Examples of pattern validation are illustrated in Fig. S1. This interval-based validation approach ensures that we exclude subgroups whose *WRAcc* could have arisen by chance in random subsets

If validated in one cohort, the same rule is bootstrapped in the other to assess generalizability.

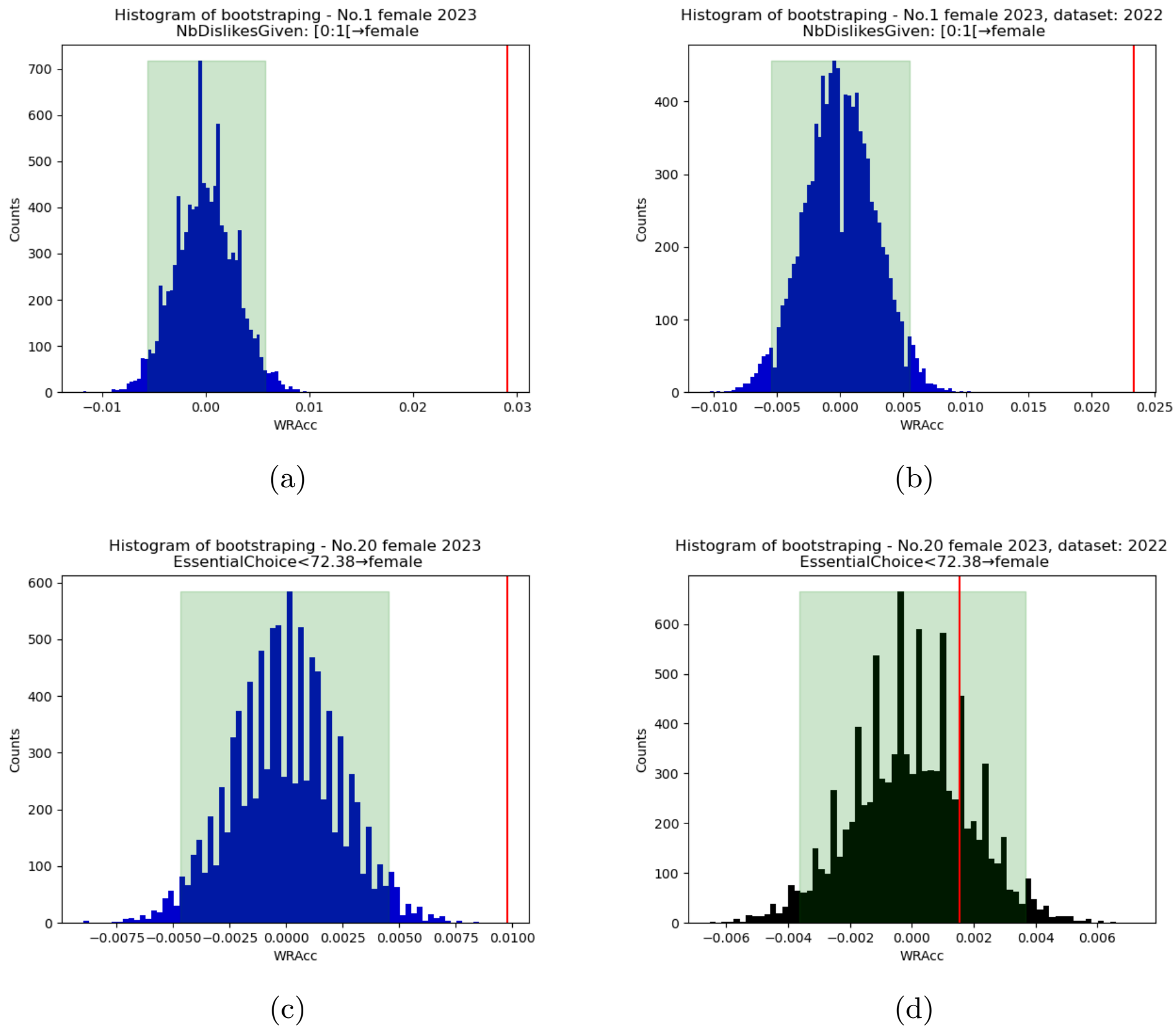

Fig. S1: Example of bootstrap validation and cross-cohort validation of subgroup patterns. The curve represents the bootstrap distribution of *WRAcc* values obtained from 10,000 random groups with the same support as the evaluated pattern. The green shaded area corresponds to the 95% confidence interval, and the vertical line indicates the *WRAcc* of the evaluated pattern. A pattern is considered validated when its *WRAcc* exceeds the upper bound of the confidence interval. Panels (a) and (c) show the validation of the first- and twentieth-ranked female patterns identified in the 2022 cohort. Both patterns are validated in their original cohort. Panels (b) and (d) show the validation of these same patterns in the 2023 cohort to assess their generalizability. The first pattern remains validated across cohorts, whereas the twentieth pattern is not validated because its *WRAcc* falls within the confidence interval.

C Inter individual variability analysis

To analyze variability, we compared individuals across rules using the *Jaccard Index (IoU)*, which quantifies the proportion of shared individuals between two subgroups. The IoU between two rules r_1 and r_2 extracted from dataset D_j is defined as:

$$Jaccard(r_1, r_2, j) = \frac{|cov(r_1) \wedge cov(r_2) \wedge D_j^t|}{|(cov(r_1) \vee cov(r_2)) \wedge D_j^t|} \tag{4}$$

D Raw Results of Subgroup Discovery and Intra- and Inter-Cohort Validation

Although *pysubgroup* reports interval selectors such as $[-1.0, 0.0[$, these intervals are equivalent to the single value -1 in our datasets because no valid observations exist between -1 and 0. Consequently, such rules identify students who did not participate in the corresponding activity rather than students with low performance.

Table S1: Extracted patterns by the *Subgroup Discovery* algorithm for the target "**female from the 2022 cohort**", indexed by their quality measure (from highest to lowest). The table highlights the rule description (column 2), its *WRAcc* value (column 3), its support (proportion of students covered by the rule; column 4), whether bootstrap validation supports this rule on the current cohort (column 5), and whether bootstrap validation supports this rule on the second cohort (column 6).

index	subgroup	WRAcc	support	validity	validity 2023
1	NbDislikesGiven: [0:1[	0.02	0,68	True	True
2	NbComments: [0:1[	0.02	0,72	True	True
3	AvgAnswer: [-1:0.0[	0.02	0,72	True	True
4	CommentsSize: [-1:5.0[	0.02	0,72	True	True
5	AvgDislikesReceived: [-1:0.0[	0.02	0,72	True	True
6	AvgLikesReceived: [-1:0.0[	0.02	0,72	True	True
7	like_given: [0:1[	0.02	0,53	True	True
8	Hepato-Gastroenterology_avg_PC: [49.72:59.92[	0.02	0,20	True	False
9	EssentialChoice: [76:80.45[	0.01	0,20	True	False
10	CorrectChoice<75.29	0.01	0,20	True	True
11	AcademicPerformance: [59.30:64.07[	0.01	0,20	True	True
12	AcademicRank: [54.43:74.77[	0.01	0,20	True	True
13	Cardiovascular_avg_PC: [13.35:56.57[	0.01	0,20	True	True
14	Nephrology_avg_IQS: [40:67.64[	0.01	0,20	True	True
15	Oncology-Radiotherapy_avg_PC: [52.21:61.12[	0.01	0,20	True	False
16	CorrectChoice: [75.29:79.10[	0.01	0,20	True	True
17	ARE_avg_PC: [37.20:55.06[	0.01	0.20	True	True
18	Internal Medicine_avg_PC: [50.26:60.19[	0.01	0,20	True	True
19	Ophthalmology_avg_PC: [42.00:60.11[	0.01	0,20	True	True
20	avg_PC_all: [53.60:59.32[	0.01	0,20	True	False

Table S2: Extracted patterns by the *Subgroup Discovery* algorithm for the target "**male from the 2022 cohort**", indexed by their quality measure (from highest to lowest). The table highlights the rule description (column 2), its *WRAcc* value (column 3), its support (proportion of students covered by the rule; column 4), whether bootstrap validation supports this rule on the current cohort (column 5), and whether bootstrap validation supports this rule on the second cohort (column 6).

index	subgroup	WRAcc	support	validity 0,20	validity 2023
1	CorrectChoice>=84.68	0.03	0,20	True	True
2	ARE_avg_dp>=67.58	0.03	0,20	True	True
3	Pneumology_avg_PC>=68.46	0.02	0,20	True	True
4	avg_PC_all>=68.50	0.02	0,20	True	True
5	EssentialChoice>=87.30	0.02	0,20	True	True
6	AcademicPerformance>=72.01	0.02	0,20	True	True
7	AcademicRank<17.21	0.02	0,20	True	True
8	Cardiovascular_avg_PC>=69.42	0.02	0,20	True	True
9	NbDislikesGiven>=4	0.02	0,20	True	True
10	Infectious Diseases_avg_PC>=68.32	0.02	0,20	True	True
11	Orthopedics_avg_PC>=67.46	0.02	0,20	True	True
12	Hepato-Gastroenterology_avg_PC>=67.21	0.02	0,20	True	True
13	AvgDislikesReceived>=0.38	0.02	0,20	True	True
14	Hematology_avg_PC>=67.15	0.02	0,20	True	True
15	NbComments>=3	0.02	0,20	True	True
16	Nephrology_avg_PC>=67.89	0.02	0,20	True	True
17	Neurology_avg_PC>=67.91	0.02	0,20	True	True
18	AvgLikesReceived>=10.22	0.02	0,20	True	True
19	Oncology-Radiotherapy_avg_PC>=68.17	0.02	0,20	True	True
20	UnacceptableChoice<10.20	0.02	0,20	True	True

Table S3: Extracted patterns by the *Subgroup Discovery* algorithm for the target "**female from the 2023 cohort**", indexed by their quality measure (from highest to lowest). The table highlights the rule description (column 2), its *WRAcc* value (column 3), its support (proportion of students covered by the rule; column 4), whether bootstrap validation supports this rule on the current cohort (column 5), and whether bootstrap validation supports this rule on the second cohort (column 6).

index	subgroup	WRAcc	support	validity	validity 2022
1	NbDislikesGiven: [0:1[	0.03	0.64	True	True
2	AvgDislikesReceived: [-1:0.0[	0.02	0.70	True	True
3	AvgLikesReceived: [-1:0.0[	0.02	0.70	True	True
4	CommentsSize: [-1:5.0[	0.02	0.70	True	True
5	NbComments: [0:1[	0.02	0.70	True	True
6	AvgAnswer: [-1:0.0[	0.02	0.70	True	True
7	Cardiovascular_avg_PC: [-1:47.18[	0.02	0.40	True	False
8	CorrectChoice<73.27	0.02	0.20	True	True
9	ARE_avg_PC: [-1.0:46.45[	0.01	0.40	True	False
10	Nephrology_avg_PC: [-1:39.58[	0.01	0.40	True	False
11	like_given: [0:1[	0.01	0.41	True	True
12	AcademicRank>=77.47	0.01	0.20	True	False
13	AcademicPerformance<65.41	0.01	0.20	True	True
14	Infectious Diseases_avg_PC: [-1:47.15[	0.01	0.40	True	False
15	nb_message: [0:1[	0.01	0.94	True	True
16	Ophthalmology_avg_PC: [1.35:50.64[	0.01	0.18	True	True
17	Hematology_avg_PC: [35.68:51.25[	0.01	0.20	True	True
18	AbandonedQuestion<4.17	0.01	0.20	True	False
19	Hepato-Gastroenterology_avg_IQS: [43.75:59.09[	0.01	0.20	True	False
20	EssentialChoice<72.38	0.01	0.20	True	False

Table S4: Extracted patterns by the *Subgroup Discovery* algorithm for the target "**Male from the 2023 cohort**", indexed by their quality measure (from highest to lowest). The table highlights the rule description (column 2), its *WRAcc* value (column 3), its support (proportion of students covered by the rule; column 4), whether bootstrap validation supports this rule on the current cohort (column 5), and whether bootstrap validation supports this rule on the second cohort (column 6).

index	subgroup	WRAcc	support	validity	validity 2022
1	NbDislikesGiven>=4	0.03	0.20	True	True
2	ARE_avg_PC>=61.76	0.02	0.20	True	True
3	NbComments>=3	0.02	0.20	True	True
4	AvgDislikesReceived>=0.01	0.02	0.18	True	True
5	Cardiovascular_avg_PC>=63.15	0.02	0.20	True	True
6	AcademicPerformance>=75.56	0.02	0.20	True	True
7	AcademicRank<18.51	0.02	0.20	True	True
8	avg_PC_all>=62.59	0.02	0.20	True	True
9	Nephrology_avg_PC>=60.65	0.02	0.20	True	True
10	AvgLikesReceived>=1.82	0.02	0.20	True	True
11	CorrectChoice>=82.75	0.02	0.20	True	True
12	CommentsSize>=69.64	0.02	0.20	True	True
13	AvgAnswer>=0.01	0.02	0.16	True	True
14	Hepato-Gastroenterology_avg_PC>=60.65	0.02	0.20	True	True
15	EssentialChoice>=84.13	0.02	0.20	True	True
16	Pneumology_avg_PC>=61.35	0.02	0.20	True	True
17	Neurology_avg_PC>=61.77	0.02	0.20	True	True
18	Urology_avg_PC>=60.69	0.02	0.20	True	True
19	Orthopedics_avg_PC>=61.77	0.02	0.20	True	True
20	Rheumatology_avg_PC>=61.43	0.02	0.20	True	True

E Overlap between engagement- and performance-related subgroup rules

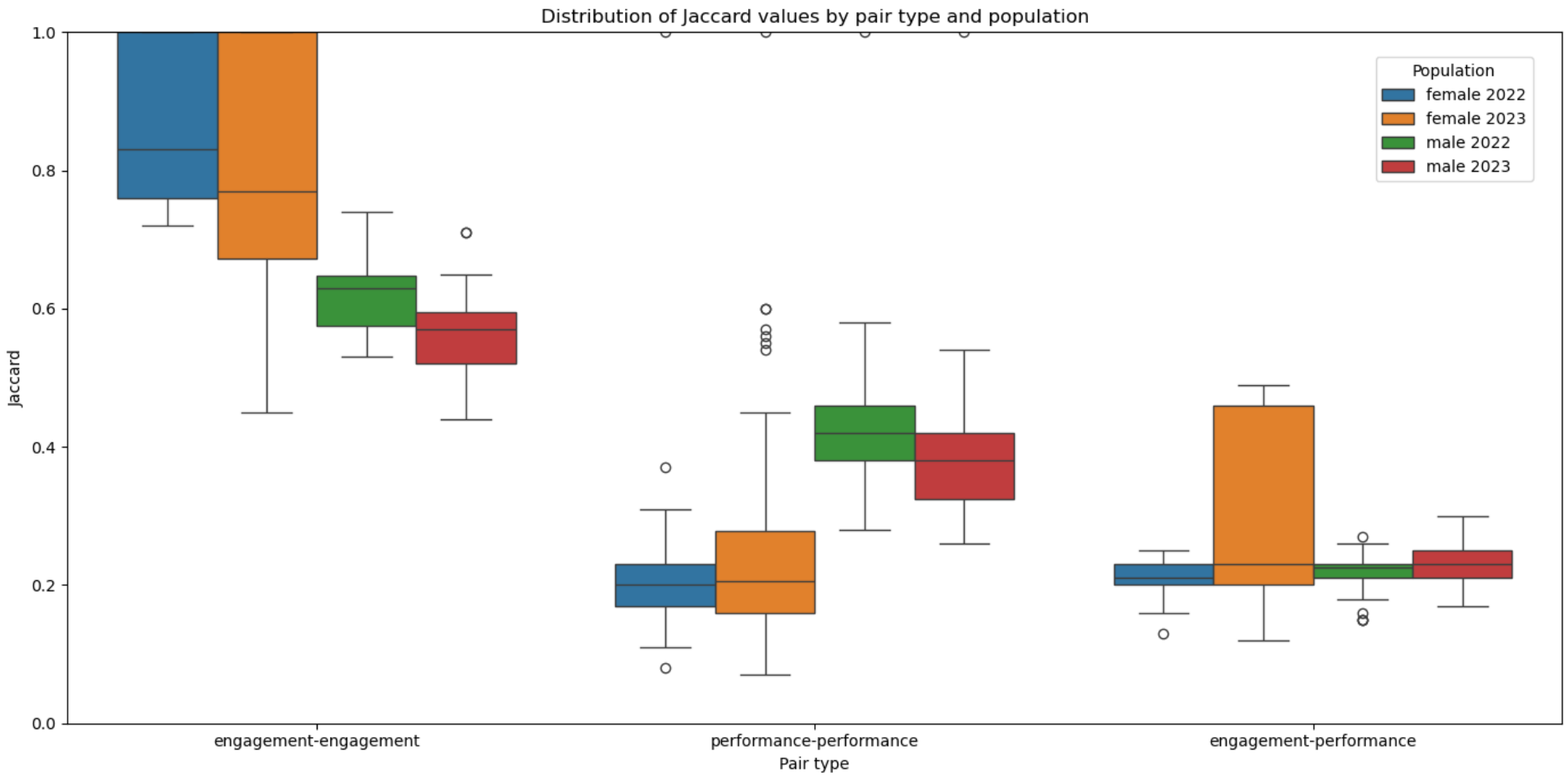

Fig. S2: Distribution of pairwise *Jaccard* indices according to rule category and cohort. Boxplots show the distribution of pairwise *Jaccard* indices between subgroups identified by engagement-related rules (engagement–engagement), performance-related rules (performance–performance), and engagement- versus performance-related rules (engagement–performance). For each rule category, distributions are presented separately for the four study populations (men 2022, men 2023, women 2022, and women 2023). The central line represents the median, the box spans the interquartile range (IQR), whiskers extend to 1.5 × IQR, and points beyond the whiskers indicate outliers. Higher *Jaccard* values indicate greater overlap between the sets of students identified by two rules.

F Descriptions of attributes studied

Table S5: Descriptive statistics by gender for the main attributes identified in the *Subgroup Discovery* analysis. The table reports the mean and standard deviation of the attributes most frequently selected in subgroup rules across cohorts and genders. Means and standard deviations were computed using only valid observations (values 0). The percentage of students with a value of -1, corresponding to the absence of activity for the considered attribute, is reported separately.

Variable	Female (no activity)	Female (mean ± SD)	Male (no activity)	Male (mean ± SD)
NbDislikesGiven	0%	5.54 ± 26.96	0%	13.45 ± 60.21
NbLikesGiven	0%	47.76 ± 184.84	0%	73.29 ± 308.01
NbComments	0%	6.04 ± 34.39	0%	11.65 ± 65.00
CommentsSize	74.1%	97.06 ± 66.08	64.5%	100.03 ± 63.45
AvgAnswer	74.1%	0.27 ± 0.38	64.5%	0.26 ± 0.35
AvgLikesReceived	74.1%	13.10 ± 21.95	64.5%	14.88 ± 24.34
AvgDislikesReceived	74.1%	1.76 ± 5.17	64.5%	2.21 ± 4.82
AcademicPerformance	0%	67.48 ± 6.87	0%	68.68 ± 7.48
AcademicRank	0%	49.09 ± 27.50	0%	43.14 ± 29.08
EssentialChoice	0.5%	79.08 ± 9.17	0.6%	80.58 ± 9.33
CorrectChoice	0.2%	78.16 ± 6.56	0.4%	79.81 ± 6.83
avg_PC_all	3.7%	57.46 ± 10.12	3.5%	59.52 ± 10.84
ARE_avg_PC	20.5%	56.43 ± 11.80	18.9%	59.07 ± 12.60
Cardiovascular_avg_PC	22.2%	58.10 ± 12.02	20.6%	60.90 ± 12.43
HG_avg_PC	31.2%	56.50 ± 12.30	30.8%	59.26 ± 12.96
Nephrology_avg_PC	29.1%	56.20 ± 12.70	27.6%	58.88 ± 13.25

Attribute	Explanation
TimeOnTask	Total time spent on quizzes before ECNp/EDNp
NbDays	Number of distinct training days
Frequency	Average interval (in days) between training sessions
Density	Number of questions attempted per training day
NbMessages	Number of messages sent via chat
NbLikesGiven	Number of likes given on student comments
NbDislikesGiven	Number of dislikes given on student comments
NbComments	Number of written comments
CommentsSize	Average length of written comments (in number of characters)
AvgAnswer	Average number of comments in response to comments written by the student
AvgDislikesReceived	Average number of dislikes received on comments written by the student
AvgLikesReceived	Average number of likes received on comments written by the student
AbandonedQuestions	Percentage of questions abandoned
IncorrectChoice	Percentage of incorrect answers
CorrectChoice	Percentage of correct answers
UnacceptableChoice	Percentage of unacceptable answers
EssentialChoice	Percentage of essential answers
EvolutionScore	Average improvement on repeated questions
AcademicRank	Student ranking at ECNp/EDNp (0–100)
AcademicPerformance	Student exam results at ECNp/EDNp (0–100)
nb_IQS_unique	Percentage of unique IQS questions attempted across all specialties
nb_IQS_all	Total number of IQS questions attempted across all specialties
avg_IQS_all	Average IQS score across all specialties (0–100)
nb_CAR_unique	Percentage of unique CAR questions attempted across all specialties
nb_CAR_tot	Total number of CAR questions attempted across all specialties
avg_CAR_all	Average CAR score across all specialties (0–100)
nb_PC_unique	Percentage of unique PC questions attempted across all specialties
nb_PC_tot	Total number of PC questions attempted across all specialties
avg_PC_all	Average PC score across all specialties (0–100)
Orthopedics_nb_IQS	Percentage of IQS questions completed in Orthopedics
Orthopedics_avg_IQS	Average IQS score in Orthopedics (0–100)
Orthopedics_nb_PC	Percentage of PC questions completed in Orthopedics
Orthopedics_avg_PC	Average PC score in Orthopedics (0–100)
Digestive Surgery_nb_IQS	Percentage of IQS questions completed in Digestive Surgery
Digestive Surgery_avg_IQS	Average IQS score in Digestive Surgery (0–100)
Digestive Surgery_nb_CAR	Percentage of CAR questions completed in Digestive Surgery
Digestive Surgery_avg_CAR	Average CAR score in Digestive Surgery (0–100)
Digestive Surgery_nb_PC	Percentage of PC questions completed in Digestive Surgery
Digestive Surgery_avg_PC	Average PC score in Digestive Surgery (0–100)
Cardiovascular_nb_IQS	Percentage of IQS questions completed in Cardiovascular
Cardiovascular_avg_IQS	Average IQS score in Cardiovascular (0–100)
Cardiovascular_nb_CAR	Percentage of CAR questions completed in Cardiovascular
Cardiovascular_avg_CAR	Average CAR score in Cardiovascular (0–100)
Cardiovascular_nb_PC	Percentage of PC questions completed in Cardiovascular
Cardiovascular_avg_PC	Average PC score in Cardiovascular (0–100)
Toxicology_nb_IQS	Percentage of IQS questions completed in Toxicology
Toxicology_avg_IQS	Average IQS score in Toxicology (0–100)
Toxicology_nb_PC	Percentage of PC questions completed in Toxicology
Toxicology_avg_PC	Average PC score in Toxicology (0–100)
Gerontology_nb_IQS	Percentage of IQS questions completed in Gerontology
Gerontology_avg_IQS	Average IQS score in Gerontology (0–100)
Gerontology_nb_CAR	Percentage of CAR questions completed in Gerontology
Gerontology_avg_CAR	Average CAR score in Gerontology (0–100)
Gerontology_nb_PC	Percentage of PC questions completed in Gerontology
Gerontology_avg_PC	Average PC score in Gerontology (0–100)

Attribute	Explanation
Urology_nb_IQS	Percentage of IQS questions completed in Urology
Urology_avg_IQS	Average IQS score in Urology (0–100)
Urology_nb_CAR	Percentage of CAR questions completed in Urology
Urology_avg_CAR	Average CAR score in Urology (0–100)
Urology_nb_PC	Percentage of PC questions completed in Urology
Urology_avg_PC	Average PC score in Urology (0–100)
ARE_nb_IQS	Percentage of IQS questions completed in Anesthesiology - Resuscitation - Emergency
ARE_avg_IQS	Average IQS score in Anesthesiology - Resuscitation - Emergency (0–100)
ARE_nb_CAR	Percentage of CAR questions completed in Anesthesiology - Resuscitation - Emergency
ARE_avg_CAR	Average CAR score in Anesthesiology - Resuscitation - Emergency (0–100)
ARE_nb_PC	Percentage of PC questions completed in Anesthesiology - Resuscitation - Emergency
ARE_avg_PC	Average PC score in Anesthesiology - Resuscitation - Emergency (0–100)
Pneumology_nb_IQS	Percentage of IQS questions completed in Pneumology
Pneumology_avg_IQS	Average IQS score in Pneumology (0–100)
Pneumology_nb_CAR	Percentage of CAR questions completed in Pneumology
Pneumology_avg_CAR	Average CAR score in Pneumology (0–100)
Pneumology_nb_PC	Percentage of PC questions completed in Pneumology
Pneumology_avg_PC	Average PC score in Pneumology (0–100)
EMN_nb_IQS	Percentage of IQS questions completed in Endocrinology - Metabolism - Nutrition
EMN_avg_IQS	Average IQS score in Endocrinology - Metabolism - Nutrition (0–100)
EMN_nb_CAR	Percentage of CAR questions completed in Endocrinology - Metabolism - Nutrition
EMN_avg_CAR	Average CAR score in Endocrinology - Metabolism - Nutrition (0–100)
EMN_nb_PC	Percentage of PC questions completed in Endocrinology - Metabolism - Nutrition
EMN_avg_PC	Average PC score in Endocrinology - Metabolism - Nutrition (0–100)
Public Health_nb_IQS	Percentage of IQS questions completed in Public Health
Public Health_avg_IQS	Average IQS score in Public Health (0–100)
Public Health_nb_CAR	Percentage of CAR questions completed in Public Health
Public Health_avg_CAR	Average CAR score in Public Health (0–100)
Public Health_nb_PC	Percentage of PC questions completed in Public Health
Public Health_avg_PC	Average PC score in Public Health (0–100)
Addictology_nb_IQS	Percentage of IQS questions completed in Addictology
Addictology_avg_IQS	Average IQS score in Addictology (0–100)
Addictology_nb_CAR	Percentage of CAR questions completed in Addictology
Addictology_avg_CAR	Average CAR score in Addictology (0–100)
Addictology_nb_PC	Percentage of PC questions completed in Addictology
Addictology_avg_PC	Average PC score in Addictology (0–100)
Gynecology-Obstetrics_nb_IQS	Percentage of IQS questions completed in Gynecology-Obstetrics
Gynecology-Obstetrics_avg_IQS	Average IQS score in Gynecology-Obstetrics (0–100)
Gynecology-Obstetrics_nb_CAR	Percentage of CAR questions completed in Gynecology-Obstetrics
Gynecology-Obstetrics_avg_CAR	Average CAR score in Gynecology-Obstetrics (0–100)
Gynecology-Obstetrics_nb_PC	Percentage of PC questions completed in Gynecology-Obstetrics
Gynecology-Obstetrics_avg_PC	Average PC score in Gynecology-Obstetrics (0–100)
Neurosurgery_nb_IQS	Percentage of IQS questions completed in Neurosurgery
Neurosurgery_avg_IQS	Average IQS score in Neurosurgery (0–100)
Neurosurgery_nb_PC	Percentage of PC questions completed in Neurosurgery
Neurosurgery_avg_PC	Average PC score in Neurosurgery (0–100)
Internal Medicine_nb_IQS	Percentage of IQS questions completed in Internal Medicine
Internal Medicine_avg_IQS	Average IQS score in Internal Medicine (0–100)
Internal Medicine_nb_PC	Percentage of PC questions completed in Internal Medicine
Internal Medicine_avg_PC	Average PC score in Internal Medicine (0–100)
Nephrology_nb_IQS	Percentage of IQS questions completed in Nephrology
Nephrology_avg_IQS	Average IQS score in Nephrology (0–100)
Nephrology_nb_CAR	Percentage of CAR questions completed in Nephrology
Nephrology_avg_CAR	Average CAR score in Nephrology (0–100)
Nephrology_nb_PC	Percentage of PC questions completed in Nephrology
Nephrology_avg_PC	Average PC score in Nephrology (0–100)
Pediatrics_nb_IQS	Percentage of IQS questions completed in Pediatrics
Pediatrics_avg_IQS	Average IQS score in Pediatrics (0–100)
Pediatrics_nb_CAR	Percentage of CAR questions completed in Pediatrics
Pediatrics_avg_CAR	Average CAR score in Pediatrics (0–100)
Pediatrics_nb_PC	Percentage of PC questions completed in Pediatrics
Pediatrics_avg_PC	Average PC score in Pediatrics (0–100)
Psychiatry_nb_IQS	Percentage of IQS questions completed in Psychiatry
Psychiatry_avg_IQS	Average IQS score in Psychiatry (0–100)
Psychiatry_nb_CAR	Percentage of CAR questions completed in Psychiatry
Psychiatry_avg_CAR	Average CAR score in Psychiatry (0–100)
Psychiatry_nb_PC	Percentage of PC questions completed in Psychiatry
Psychiatry_avg_PC	Average PC score in Psychiatry (0–100)

Attribute	Explanation
Otorhinolaryngology_nb_IQS	Percentage of IQS questions completed in Otorhinolaryngology
Otorhinolaryngology_avg_IQS	Average IQS score in Otorhinolaryngology (0–100)
Otorhinolaryngology_nb_CAR	Percentage of CAR questions completed in Otorhinolaryngology
Otorhinolaryngology_avg_CAR	Average CAR score in Otorhinolaryngology (0–100)
Otorhinolaryngology_nb_PC	Percentage of PC questions completed in Otorhinolaryngology
Otorhinolaryngology_avg_PC	Average PC score in Otorhinolaryngology (0–100)
HG_nb_IQS	Percentage of IQS questions completed in Hepato-Gastroenterology
HG_avg_IQS	Average IQS score in Hepato-Gastroenterology (0–100)
HG_nb_CAR	Percentage of CAR questions completed in Hepato-Gastroenterology
HG_avg_CAR	Average CAR score in Hepato-Gastroenterology (0–100)
HG_nb_PC	Percentage of PC questions completed in Hepato-Gastroenterology
HG_avg_PC	Average PC score in Hepato-Gastroenterology (0–100)
Ophthalmology_nb_IQS	Percentage of IQS questions completed in Ophthalmology
Ophthalmology_avg_IQS	Average IQS score in Ophthalmology (0–100)
Ophthalmology_nb_CAR	Percentage of CAR questions completed in Ophthalmology
Ophthalmology_avg_CAR	Average CAR score in Ophthalmology (0–100)
Ophthalmology_nb_PC	Percentage of PC questions completed in Ophthalmology
Ophthalmology_avg_PC	Average PC score in Ophthalmology (0–100)
Hematology_nb_IQS	Percentage of IQS questions completed in Hematology
Hematology_avg_IQS	Average IQS score in Hematology (0–100)
Hematology_nb_CAR	Percentage of CAR questions completed in Hematology
Hematology_avg_CAR	Average CAR score in Hematology (0–100)
Hematology_nb_PC	Percentage of PC questions completed in Hematology
Hematology_avg_PC	Average PC score in Hematology (0–100)
OR_nb_IQS	Percentage of IQS questions completed in Oncology-Radiotherapy
OR_avg_IQS	Average IQS score in Oncology-Radiotherapy (0–100)
OR_nb_CAR	Percentage of CAR questions completed in Oncology-Radiotherapy
OR_avg_CAR	Average CAR score in Oncology-Radiotherapy (0–100)
OR_nb_PC	Percentage of PC questions completed in Oncology-Radiotherapy
OR_avg_PC	Average PC score in Oncology-Radiotherapy (0–100)
PMR_nb_IQS	Percentage of IQS questions completed in Physical Medicine and Rehabilitation
PMR_avg_IQS	Average IQS score in Physical Medicine and Rehabilitation (0–100)
PMR_nb_PC	Percentage of PC questions completed in Physical Medicine and Rehabilitation
PMR_avg_PC	Average PC score in Physical Medicine and Rehabilitation (0–100)
ID_nb_IQS	Percentage of IQS questions completed in Infectious Diseases
ID_avg_IQS	Average IQS score in Infectious Diseases (0–100)
ID_nb_CAR	Percentage of CAR questions completed in Infectious Diseases
ID_avg_CAR	Average CAR score in Infectious Diseases (0–100)
ID_nb_PC	Percentage of PC questions completed in Infectious Diseases
ID_avg_PC	Average PC score in Infectious Diseases (0–100)
Rheumatology_nb_IQS	Percentage of IQS questions completed in Rheumatology
Rheumatology_avg_IQS	Average IQS score in Rheumatology (0–100)
Rheumatology_nb_CAR	Percentage of CAR questions completed in Rheumatology
Rheumatology_avg_CAR	Average CAR score in Rheumatology (0–100)
Rheumatology_nb_PC	Percentage of PC questions completed in Rheumatology
Rheumatology_avg_PC	Average PC score in Rheumatology (0–100)
Dermatology_nb_IQS	Percentage of IQS questions completed in Dermatology
Dermatology_avg_IQS	Average IQS score in Dermatology (0–100)
Dermatology_nb_CAR	Percentage of CAR questions completed in Dermatology
Dermatology_avg_CAR	Average CAR score in Dermatology (0–100)
Dermatology_nb_PC	Percentage of PC questions completed in Dermatology
Dermatology_avg_PC	Average PC score in Dermatology (0–100)
Maxillofacial Surgery_nb_IQS	Percentage of IQS questions completed in Maxillofacial Surgery
Maxillofacial Surgery_avg_IQS	Average IQS score in Maxillofacial Surgery (0–100)
Maxillofacial Surgery_nb_PC	Percentage of PC questions completed in Maxillofacial Surgery
Maxillofacial Surgery_avg_PC	Average PC score in Maxillofacial Surgery (0–100)
Forensic Medicine_nb_IQS	Percentage of IQS questions completed in Forensic Medicine
Forensic Medicine_avg_IQS	Average IQS score in Forensic Medicine (0–100)
Forensic Medicine_nb_PC	Percentage of PC questions completed in Forensic Medicine
Forensic Medicine_avg_PC	Average PC score in Forensic Medicine (0–100)
Neurology_nb_IQS	Percentage of IQS questions completed in Neurology
Neurology_avg_IQS	Average IQS score in Neurology (0–100)
Neurology_nb_CAR	Percentage of CAR questions completed in Neurology
Neurology_avg_CAR	Average CAR score in Neurology (0–100)
Neurology_nb_PC	Percentage of PC questions completed in Neurology
Neurology_avg_PC	Average PC score in Neurology (0–100)

**Attribute**	**Explanation**
Pharmacologie-Thérapeutique_nb_IQS	Percentage of IQS questions completed in Pharmacology and Therapeutics
Pharmacologie-Thérapeutique_avg_IQS	Average IQS score in Pharmacology and Therapeutics (0–100)
Pharmacologie-Thérapeutique_nb_CAR	Percentage of CAR questions completed in Pharmacology and Therapeutics
Pharmacologie-Thérapeutique_avg_CAR	Average CAR score in Pharmacology and Therapeutics (0–100)
Pharmacologie-Thérapeutique_nb_PC	Percentage of PC questions completed in Pharmacology and Therapeutics
Pharmacologie-Thérapeutique_avg_PC	Average PC score in Pharmacology and Therapeutics (0–100)
Occupational Medicine_nb_IQS	Percentage of IQS questions completed in Occupational Medicine
Occupational Medicine_avg_IQS	Average IQS score in Occupational Medicine (0–100)
Occupational Medicine_nb_CAR	Percentage of CAR questions completed in Occupational Medicine
Occupational Medicine_avg_CAR	Average CAR score in Occupational Medicine (0–100)
Occupational Medicine_nb_PC	Percentage of PC questions completed in Occupational Medicine
Occupational Medicine_avg_PC	Average PC score in Occupational Medicine (0–100)
University	University where the student is enrolled
Region	Geographical region of France where the student's university is located
Gender	Student's gender

Table S6: Complete list of the attributes included in the analyses, together with their descriptions.
